# A novel angiotensin(1-7) agonist, PNA5, reduces ischemic reperfusion injury and cardiac dysfunction

**DOI:** 10.3389/fcvm.2026.1769276

**Published:** 2026-03-06

**Authors:** Christina Hoyer-Kimura, Meredith Hay, Methawasin Methajit, Robin Polt, Victoria Salcedo, Joshua P. Fricks, Arian Piepho, Marissa Lopez-Pier, Maricela Pier, Vito A. Marino, John P. Konhilas

**Affiliations:** 1 Department of Physiology, The University of Arizona, Tucson, AZ, United States; 2 Evelyn F. McKnight Brain Institute, The University of Arizona, Tucson, AZ, United States; 3 ProNeurogen, Inc., Tucson, AZ, United States; 4 Department of Cellular and Molecular Medicine, The University of Arizona, Tucson, AZ, United States; 5 Department of Chemistry and Biochemistry, The University of Arizona, Tucson, AZ, United States; 6 College of Nursing, The University of Arizona, Tucson, AZ, United States

**Keywords:** angiotensin-(1-7) [Ang-(1-7)], fibrosis, inflammation, ischemic reperfusion injury, PNA5, speckle echocardiography

## Abstract

**Introduction:**

Ischemic heart disease, typically caused by myocardial infarction (MI), is the leading cause of death. Ischemic reperfusion (IR) injury following MI is multifaceted, driven by reactive oxidative species (ROS), calcium overload, and inflammatory responses. Because our novel glycopeptide derivative of Angiotensin-(1-7), PNA5, has an improved half-life, decreases circulating inflammatory cytokines, and inhibits endothelial ROS production, we predict that PNA5 will attenuate IR sequelae post-IR.

**Methods:**

Three-month-old C57Bl/6J male mice were subjected to IR and treated subcutaneously, with PNA5 (100 µg/kg/day, *n* = 14) or saline (*n* = 12) starting immediately after reperfusion and continued daily for 8 weeks. Echocardiograms were taken 2, 5, and 8 weeks post-IR in B-mode using the Vevo 2100 High-Resolution Imaging System (Visual Sonics, Canada). Data were analyzed using Vevo 2100® analytic software. The hearts of the mice were stained for infarct size using 2-3-4-triphenyltetrazolium chloride, fibrosis using Picrosirius Red, and inflammation via immunofluorescence for TNFα.

**Results:**

Conventional transthoracic echocardiography showed early improvement in ejection fraction by 5 weeks post-IR. Using speckle echocardiography, we demonstrated that PNA5 treatment improved the parameters of strain and dyssynchrony in regional and temporal domains. Global longitudinal strain (GLS) and left ventricular dyssynchrony showed continued dysfunction in untreated animals post-IR, whereas measures of ejection fraction did not. Along with reduced infarct size, PNA5 treatment improved cardiac remodeling, evidenced by reduced scarring within the midapical regions of the heart compared with untreated animals.

**Conclusion:**

These data suggest that PNA5 treatments improve heart outcomes post-IR and could potentially be a therapeutic for IR injury; measures of GLS and dyssynchrony may provide more relevant insight into the protective effects of PNA5.

## Introduction

Ischemic heart disease remains the leading causes of death globally (1). Coronary artery disease and myocardial infarction (MI) are the primary underlying causes of ischemic heart disease and represent over 50% of all-cause cardiovascular disease (CVD) morbidities, which increases even more with age (2–4). To this day, early revascularization after MI remains the most effective strategy to salvage ischemic myocardium, minimize cardiac damage, and prevent heart failure (HF) (5–7). However, revascularization, or reperfusion, of ischemic cardiac tissue exacerbates cardiac injury early on. Ischemic reperfusion (IR) injury is multifaceted and includes proliferation of reactive oxidative species (ROS), calcium overload, and inflammatory responses, all contributing to increased damage (1, 8). Further loss of myocardium from IR injury results in more extensive scarring, metabolic alterations, and more extreme morphological changes, such as wall thinning and stiffening, and disruption of synchronized wall contractions. Currently, there are limited to no available therapies that attenuate IR injury, suppress IR injury inflammation, and stimulate beneficial post-IR cardiac function and repair.

Detection of subacute cardiac abnormalities prior to the development of classical HF symptoms is essential to mitigating irreversible heart damage, identifying individuals at risk, and understanding preventive therapies for IR injury. Two-dimensional speckle tracking with strain analysis provides non-invasive indices to determine subacute myocardial dysfunction with greater accuracy than ejection fraction (EF) (9, 10). Previous studies indicate that global longitudinal strain (GLS) is a more sensitive prognostic indicator than left ventricular EF for predicting clinical outcomes and pathological remodeling in patients with acute MI (11). Strain is a measure of tissue deformation during the cardiac cycle in one dimension, which is typically homogeneously distributed in healthy, non-ischemic myocardium (9, 12). Considering the localized nature of ischemic cardiac tissue following MI, strain analysis provides a sensitive means for detecting not only defects in GLS, but also regional myocardial dysfunction. For example, strain analysis is strongly correlated to measures of cardiac function and objectively predicts alterations in wall morphology, such as scarring and increased fibrosis, a key characteristic of IR injury (13).

Angiotensin-(1-7) (Ang-(1-7)) exerts its biological effects primarily through activation of the Mas receptor (MasR), which was first identified as a functional Ang-(1-7) receptor through seminal studies by Santos and Walther and their colleagues (14–16). These foundational investigations established MasR as a critical mediator of Ang-(1-7)-dependent cardiovascular signaling, including vasodilation, antifibrotic actions, and protection against pathological remodeling. Mas receptor (MasR) activation via Ang-(1-7) imparts cardioprotective effects by decreasing ROS, inhibiting proinflammatory cytokine production and fibrosis, and promoting vasodilation (17–19). Furthermore, increases in Ang-(1-7) activity and concentration with angiotensin receptor blockers improve EF post-IR (20).

Our research team has developed, and pharmacologically optimized, PNA5, a novel synthetic glycopeptide derivative of Ang-(1-7), that has an improved half-life, decreases circulating inflammatory cytokines 8 weeks post-IR, and inhibits endothelial ROS production. In addition, we have established that PNA5 and Ang-(1-7) do not negatively affect cardiac function or cardiac morphology in healthy animals (17). Considering the cardioprotective properties of Ang-(1-7) and validation that PNA5 acts as a MasR ligand, we predicted that PNA5 will attenuate IR injury and IR injury sequelae post-IR compared with untreated control animals. The purpose of the present study is to establish whether PNA5 treatment improves cardiac function and reduces infarction size in IR hearts 8 weeks after insult. We further aim to identify the effects of PNA5 on left ventricular segments using strain and fibrosis analyses to determine the regional effects on cardiac function. To overcome the limitations of using EF as a measure of systolic function such as load and heart rate dependence, we employ strain analysis using speckle tracking–based echocardiography (STE) to improve the sensitivity and specificity of how PNA5 treatment impacts IR injury.

With these aims in mind, we hypothesize that PNA5 treatment reduces IR-related infarct size and preserves cardiac function. To test these hypotheses, longitudinal echocardiographic strain analyses were performed in conjunction with histological and inflammatory assessments in a mouse model of ischemia–reperfusion injury. Although Ang-(1-7) has already shown therapeutic potential for preventing IR injury (21, 22), we are the first to present evidence of how PNA5, an MasR agonist, positively impacts infarct size with simultaneous global and regional strain parameters post-IR.

## Methods

### Animals

#### Surgical procedures

An ischemic-reperfusion protocol was utilized in this study. Mice were anesthetized, intubated, and ventilated with 2.5% isoflurane in a mixture of air and O_2_. A single injection of Buprenorphine-SR (Reckitt Benckiser Healthcare, Slough, UK) at 1 mg/kg body weight was administered prior to surgery. A left anterior thoracotomy was performed to expose the heart to visualize the left coronary artery (LCA). The LCA was occluded; ligation was confirmed by observing myocardial blanching of the left ventricular anterior wall and apex and T-wave elevation. Following 45 min of occlusion, the ligature was released, restoring blood flow to the previously ischemic region (23, 24).

#### Experimental design

All animal use in this study conformed to the guidelines of the Institutional Animal Care and Use Committee at the University of Arizona and the National Institutes of Health Guidelines for the Care and Use of Laboratory Animals. Mice (*n* = 3 per cage) were housed in a standard facility that was temperature- and humidity-controlled. The facility maintained a 12-h light/dark cycle. For the duration of the study, mice had *ad libitum* access to water and standard chow. In previously published studies, we have shown that treatments with daily injections of Ang-(1-7) and PNA5 have no negative effects on heart function or circulating cytokines in both sham mice and in mice with already established chronic HF (17, 19).

**Figure 1:**
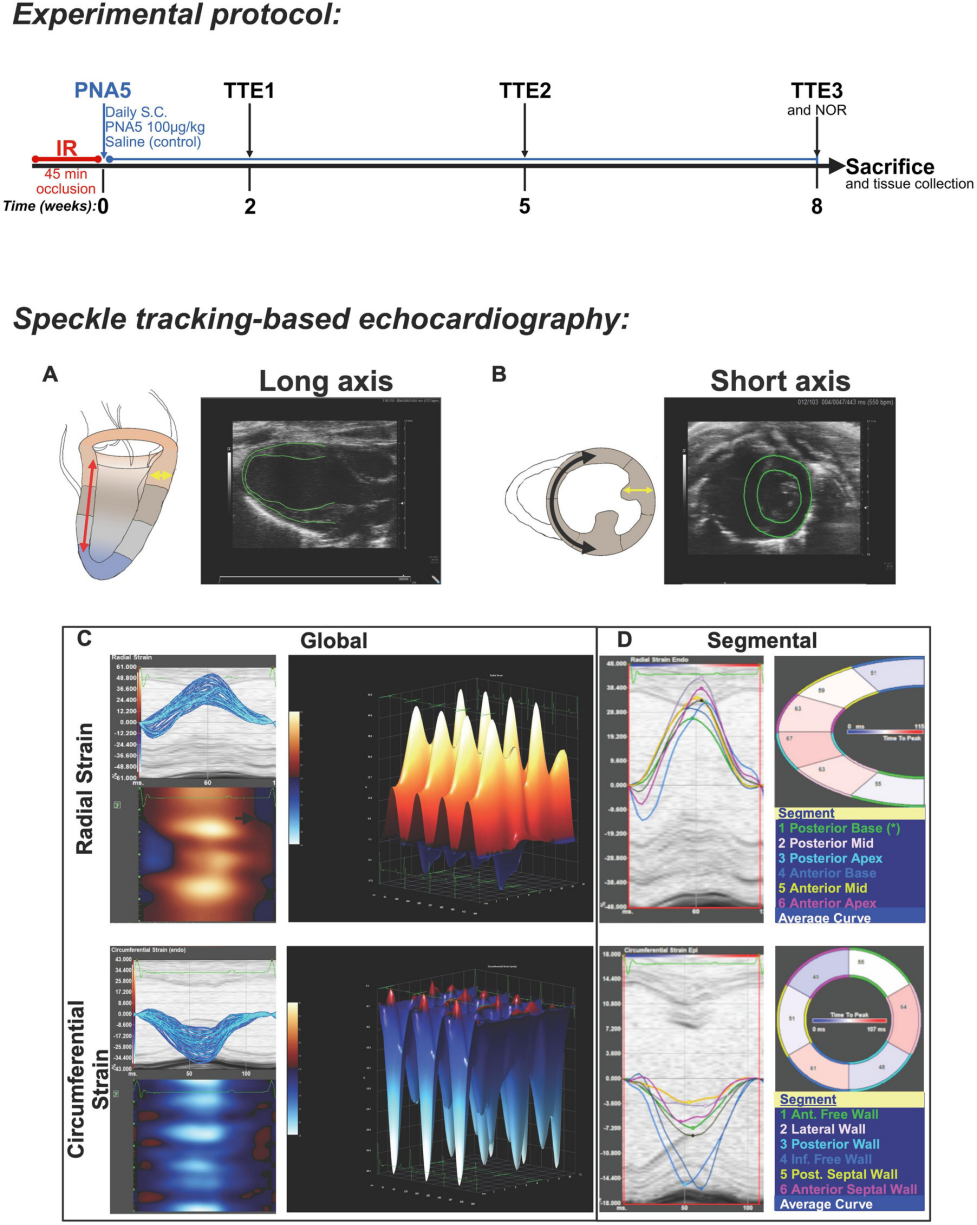
Experimental design and methods. Top panel. An experimental protocol where mice were subjected to an ischemic reperfusion (IR) protocol, immediately followed by the administration of PNA5 (100 µg/kg mouse weight subcutaneously) or saline continued daily for 8 weeks. Transthoracic echocardiography (TTE) with two-dimensional speckle tracking images was taken at 2 weeks (TTE1), 5 weeks (TTE2), and 8 weeks (TTE3) following IR injury. A total of *n* = 15 mice underwent IR surgery. **(A)** A cartoon of cardiac parasternal long-axis view (PLAX) adjacent to a representative 2D PLAX TTE image with LV trace in green. The following colors and areas of the heart are listed: tan = base, gray = mid, and blue = apex. The red arrow represents longitudinal strain during systole. The yellow arrow represents radial strain measured in the long-axis view. **(B)** A cartoon of cardiac short-axis view at the mid region adjacent to a representative 2D TTE image with LV trace in green. The black arrow represents circumferential strain during systole. The yellow arrow represents radial strain measured in the short-axis view. **(C)** Left panel: Global radial and circumferential strain illustrated as percent changes in LV wall deformation during one cardiac cycle over time (blue trace) or heatmap (blue/orange); right panel: 3D strain deformation shows an increase in distance during systole (orange/positive values) and a decrease in distance during systole (blue/negative values) over four consecutive cardiac cycles. **(D)** Representative LV radial and circumferential strain curves (six LV segments color-coordinated with the LV segment) and time-to-peak curves.

Male C57Bl/6J mice (Jackson Laboratories, Bar Harbor, ME, USA) were subject to myocardial IR injury protocol at 3 months of age. Upon conclusion of the IR protocol (while mice were still anesthetized), the mice received their first dose of PNA5 (*n* = 14) at 100 µg/kg/day or saline (*n* = 12). PNA5 or saline was subsequently delivered daily via subcutaneous injections for 8 weeks post-IR (Figure 1). An 8-week timeline was established by supporting previous heart failure studies where mice underwent permanent LCA ligation followed by daily PNA5 treatments. Treatment groups were randomly preassigned to animals prior to surgery in order to deter bias. The mice underwent transthoracic echocardiography (TTE) at 2, 5, and 8 weeks post-IR surgery. At 8 weeks post-IR, the mice also underwent a novel object recognition test (NOR) and were then sacrificed. Control mice that underwent sham surgery were treated with saline (*n* = 5), dimethyl sulfoxide (DMSO) (*n* = 5), or PNA5 (*n* = 6) s.c. (Supplementary Methods). At 8 weeks post-IR, the mice were anesthetized in an induction chamber with 5% isoflurane in a mixture of air and O_2_. Loss of the pedal withdrawal reflex, confirmed by toe pinch, was used to check for full sedation, and the mice were euthanized via cervical dislocation in accordance with institutional animal care and use guidelines. The hearts of the mice were then promptly excised and further processed for staining.

### Conventional and speckle tracking–based strain echocardiography

#### Transthoracic echocardiography

TTE were performed 2 weeks (TTE1), 5 weeks (TTE2), and 8 weeks (TTE3) post-IR. Echocardiographs were taken on anesthetized mice (1.5%–2.0% isoflurane) using the Vevo 2100 High-Resolution Imaging System (Visual Sonics, Toronto, ON, Canada) with a 25-MHz transducer. The heart rate was maintained between 450 and 530 beats/min. Echocardiographic images were taken in B mode. Data from blinded treatment groups were analyzed using Vevo 2100® analytic software (Visual Sonics, Toronto, ON, Canada) and Vevo Strain (Visual Sonics, Toronto, ON, Canada). The results were an average of three measured values per animal.

#### Speckle tracking–based echocardiograph

To ensure repeatability and quality control of speckle tracking–based strain measurements, echocardiographic acquisitions were performed using standardized imaging protocols with consistent transducer positioning, imaging planes, and anesthesia conditions across animals and time points. The heart rate was maintained at 450–530 beats/min to minimize variability related to loading conditions. Strain analyses were performed using the same software platform (Vevo Strain, Vevo LAB v1.7.1) and standardized segmentation models for long-axis and short-axis views. Measurements were averaged across three consecutive cardiac cycles for each animal. Automated tracking was visually inspected, and segments with inadequate tracking quality were excluded from analyses. All analyses were conducted by investigators blinded to treatment group.

Strain imaging using the STE of three cycles from the parasternal long (Figure 1A) and short axes (Figure 1B) was executed for strain analysis of the left ventricle (LV) using Vevo Strain Software (Vevo LAB 1.7.1). Strain was calculated either in the longitudinal axis (apex to base, red arrow; Figure 1A), radial axis (LV cavity to LV wall, yellow arrows; Figures 1A,B), or circumferential axis (endocardial shortening, black arrow; Figure 1B); radial strain was measured from the long and short axes and circumferential strain was measured from the short axis at the apex, mid, and base levels. Because myocardial motion occurs in three dimensions, strain was quantified along longitudinal, radial, and circumferential axes to comprehensively assess LV function. Longitudinal strain reflects myocardial shortening along the apex-to-base axis (red arrow, Figure 1A) and is expressed as a negative value during systole as the apex moves toward the base of the heart. Radial strain quantifies myocardial wall thickening during systole (yellow arrows, Figures 1A,B) and is expressed as a positive value. Circumferential strain reflects endocardial shortening around the LV circumference (black arrow, Figure 1B) and was measured from short-axis views at the apex, mid, and base levels.

Strain is represented as a percent fractional length change from the initial length during myocardial systolic deformation (9, 12). Strain appears positive when there is an increase in length or negative when there is a decrease in length. Representative traces of an individual cardiac cycle from the global radial (Figure 1C, left top panel) and the circumferential (Figure 1C, left bottom panel) axes show % relative strain over time in a line plot. The line plot can be interpolated into a heatmap that shows strain via color over time (*x*-axis) per location on the left ventricle (*y*-axis). The heatmap image can be further modified into a three-dimensional (3D) figure that shows strain over time (ms) across the heart for multiple cardiac cycles (Figure 1C, middle top panel—radial strain, middle bottom panel—circumferential strain, and longitudinal). In both the heatmap and the 3D figures, strain deformation during systole causing an increase in length is represented in orange, and a decrease in length is represented in blue.

Global longitudinal strain (GLS) was calculated as the average peak longitudinal strain across all left ventricular segments and served as a global index of myocardial systolic function. Strain within the segments of the heart can also be analyzed independently (Figure 1D). From the long-axis view (Figure 1D, top panel), the six LV segments are the posterior base, posterior mid, posterior apex, anterior base, anterior mid, and anterior apex. The short-axis six LV segments (Figure 1D, bottom panel) are the anterior free wall, lateral free wall, posterior wall, inferior free wall, posterior septal wall, and anterior septal wall. Strain for each of the six LV segments is represented in a coordinated line plot that shows strain over time in the cardiac cycle. Peak strain magnitude (PK%) was defined as the maximum absolute strain value reached during systole for each cardiac cycle and was used as a measure of myocardial contractile function. These values were used to determine strain segmentally and were also included in the calculations for dyssynchrony.

Dyssynchrony measures the heart's functional movement in one wall compared with another; it represents the heart's ability to move uniformly during a contraction (25, 26), LV mechanical dyssynchrony was determined from both long-axis and short-axis views. Dyssynchrony was calculated using two methods. (1) Dyssynchrony was calculated via the maximum time-delay-to-peak systolic strain (Time to Peak; T2P) between the opposing wall segments of the six LV segments (Time to Peak; ΔT2P) showing opposing wall dyssynchrony. Dyssynchrony in the long axis included opposing wall segments (anterior/posterior) at the apex, mid, and base. Dyssynchrony in the short axis included the following opposing wall segments at the apex, mid, and base: anterior and inferior free walls (anterior and posterior segments), anterior and inferior walls (anterolateral and inferolateral segments), and anterior and inferior septal walls (anteroseptal and inferoseptal segments). (2) Dyssynchrony was calculated using the standard deviation of T2P (STD T2P), showing regional wall dyssynchrony at the apex, mid, and base, providing a measure of overall temporal dispersion of myocardial contraction. Together, these complementary dyssynchrony metrics characterize both opposing-wall delay and regional heterogeneity in contraction timing across the LV.

### Infarct size determination, histopathology, and immunofluorescence

#### Infarct size

Upon sacrifice, the LCA was religated, and the abdominal aorta was clamped. Evans Blue (0.75%; Sigma, St. Louis, MO, USA) dye was introduced into the LV apex to distinguish the area at risk (AAR) within the LV. The heart was immediately excised and frozen at −20°C for 20 min and transversely sectioned (1 mm thick). The heart sections were incubated in 1% 2,3,4-triphenyltetrazolium chloride stain (TTC; Sigma) for 30 min at 37°C in a water bath to identify the infarct area (white) from viable tissue (red). The area of the infarct and LV was measured using ImageJ. The infarct area was measured in pixels. The values for both the infarct area and the infarct area normalized to the LV were evaluated. Normalizing infarct size to total LV area was performed to account for interanimal variability in heart size and to assess infarct burden relative to the overall LV myocardium.

#### Picrosirius Red staining to determine fibrosis/collagen content

Following infarct size determination, the sectioned hearts were then fixed in 10% formalin overnight. The fixed hearts were dehydrated in methanol and paraffin-embedded and sectioned (5 μm) for immunohistochemical analysis. The paraffin-embedded hearts were stained with Picrosirius Red (PSR) (24) Wiegert's hematoxylin (1% hematoxylin and 5%EtOH), followed by PSR (1% concentrated HCl and 1.16% ferric chloride). Full images of each heart section were stitched from images taken using Lecia DMI6000 for both bright field and polarized (exposure time 125 ms) settings at 20X.

Collagen content was quantified from bright field and polarized images using ImageJ; Color Threshold ImageJ function was used to determine the total tissue area and collagen area in the region of interest (ROI). ROIs were assigned according to a standardized 17-segment left ventricular model adapted from clinical infarct mapping, spanning the basal, mid, and apical regions (27). The ROIs were taken from the infarct, the superficial myocardium, and the mid myocardium in each area of the left ventricle segmentation (1- Basal Anterior, 2-Basal Anteroseptal, 3-Basal Inferoseptal, 4-Basal Inferior, 5-Basal Inferolateral, 6-Basal Anterolateral, 7-Mid Anterior, 8-Mid Anteroseptal, 9-Mid Inferoseptal, 10-Mid Inferior, 11-Mid Inferolateral, 12-Mid Anterolateral, 13-Apical Anterior, 14-Apical Septal, 15-Apical Inferior, 16-Apical Lateral, 17-Apex). Collagen inlay was measured as the area of collagen (polarized image) to the corresponding total tissue area (bright field image) in each ROI. To determine the average collagen inlay per segment, the ROI values from the infarct (if there were infarct), superficial myocardium, and mid myocardium were averaged per segment. Individual infarct collagen inlay was also analyzed independently.

Segmental analyses were performed to characterize the spatial distribution of fibrosis following IR injury and to assess the regional effects of PNA5 treatment within the infarct-associated and peri-infarct myocardium. Whole-LV segmentation additionally enabled the evaluation of remote myocardial regions to assess potential off-target or adverse fibrotic remodeling. Segments containing infarction are indicated in Supplementary Table S1.

#### Immunofluorescent staining and tyramide signal amplification

The heart sections were stained with TNFα (1:500, 17590-1AP, Proteintech, Rosemont, IL) (Rabbit, IgG 1:100), or HIF1α (1:500, PA1-16601, Invitrogen, Waltham, MA, USA) (Rabbit, IgG 1:100). Further description of the stained sections can be found in the Supplementary Methods.

### Novel object recognition test

Mice cognition was measured using a NOR test as described previously (17–19). For more detailed methods, see Supplementary Methods. There were no differences between groups for the familiarization phase, indicating that exploration was not impaired between groups.

### Statistics

GraphPad Prism was used to analyze data, and values were represented as mean ± SEM. Outliers two standard deviations away from the mean were excluded prior to other statistical analyses. All datasets were tested for normal Gaussian distribution using the Shapiro–Wilk test as it is an appropriate test for group sizes smaller than 50. All measurements were executed by blinded observers, including echocardiography, histopathology, and immunofluorescence. Each biological sample included the averaged values of all technical replicates.

To determine the impact of the IR protocol with or without PNA5 treatment over time, we employed a two-way repeated measures ANOVA followed by multiple comparisons to determine the differences between each experimental group and time point. For primary functional outcomes [EF, end-diastolic volume (EDV), end-systolic volume (ESV), GLS, and pk%], *post hoc* comparisons between experimental groups and time points were performed using Fisher's least significant difference (LSD). Because T2P was assessed at multiple anatomical levels (base, mid, and apex) and walls (free wall and septal wall), we controlled the false discovery rate across these related endpoints using the two-stage Benjamini–Krieger–Yekutieli procedure (*Q* = 0.05).

Infarct measures, fibrosis, TNFα, and HIf1α were tested for significance via unpaired Student's *t*-tests. For segmental fibrosis, analyses were guided by predefined anatomical segmentation in relation to infarct distribution. Segmental fibrosis comparisons between saline- and PNA5-treated hearts were performed using unpaired Student's *t*-tests for predefined LV segments. Comparisons between treatment groups were hypothesis-driven for the infarct-associated and peri-infarct regions, while whole-LV mapping was used descriptively to assess the spatial patterns of fibrosis and potential remote remodeling. Accordingly, formal correction for multiple comparisons was not applied.

The associations between GLS, inflammation, TNFα, and fibrosis were analyzed using simple linear regression. The best-fit line for correlations was generated via linear regression and Pearson's correlation, among others.

## Results

### PNA5 improves ejection fraction post-IR

**Figure 2:**
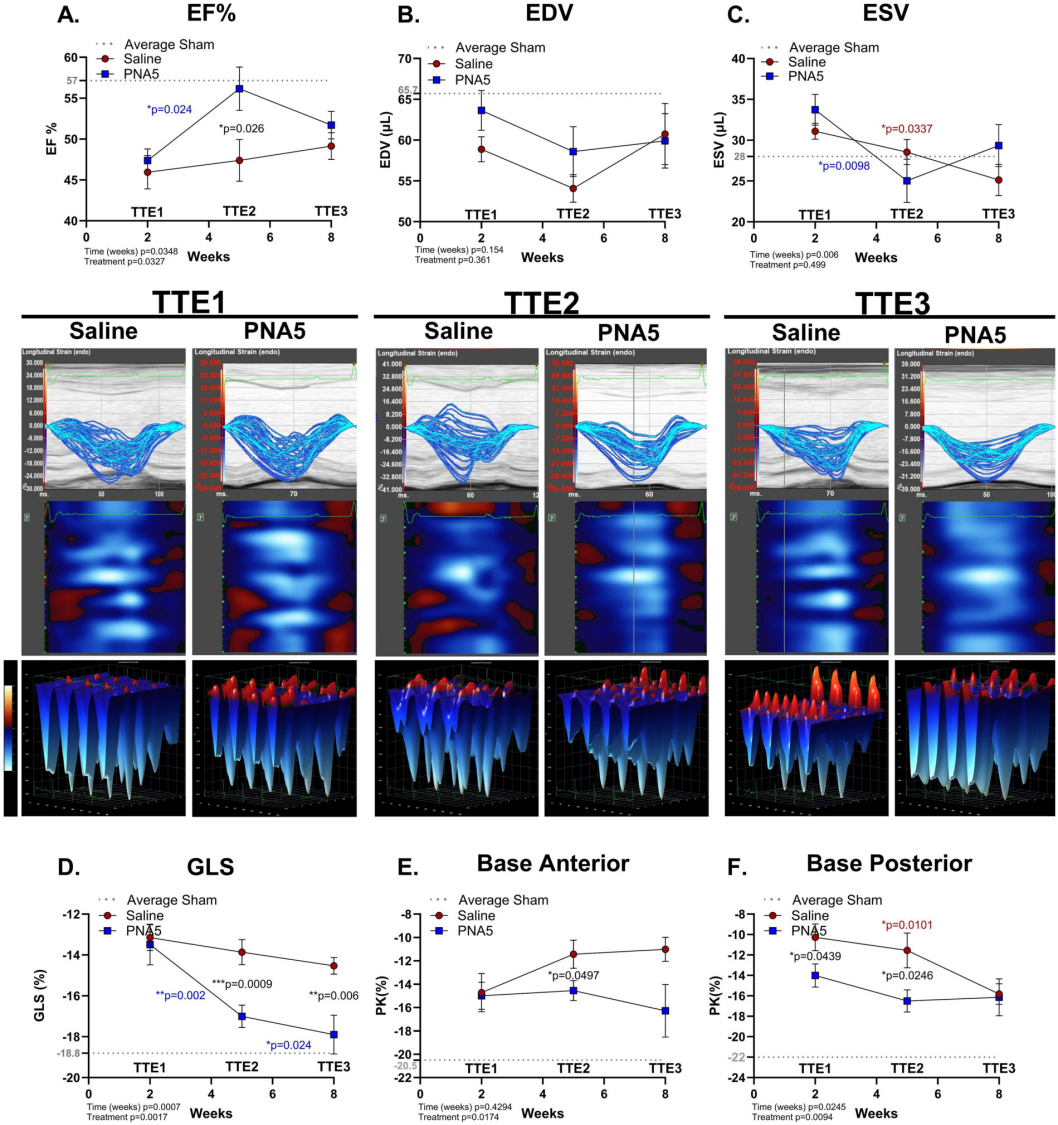
Conventional echocardiography and PLAX STE strain analysis. Conventional PLAX transthoracic echocardiography (TTE) at 2 weeks (TTE1), 5 weeks (TTE2), and 8 weeks (TTE3) post-IR: **(A)** ejection fraction (EF%), **(B)** end-diastolic volume (EDV), and **(C)** end-systolic volume (ESV). For each graph **(A–C)**, the mean baseline sham value is indicated as the dotted gray line for **(A)** EF%: 57.2, **(B)** EDV: 65.7, and **(C)** ESV: 28.4. STE strain analysis (middle panel): Representative images of change in LV deformation (%) over time (ms) (blue trace) with associated heatmap and 3D deformation (blue/red) along the longitudinal axis at TTE1, TTE2, and TTE3. A line plot representation of saline- (red circles) and PNA5 (blue squares)-treated IR mice at time points TTE1, TTE2, and TTE3 for **(D)** global longitudinal strain (GLS), **(E)** peak strain (PK%; the highest segmental strain value) at the base anterior and **(F)** PK% at the base posterior LV walls (saline, *n* = 12; PNA5, *n* = 13). For each graph **(D–F)**, the mean baseline sham value is indicated as the dotted gray line for: **(D)** GLS: −18.8, **(E)** PK longitudinal base anterior: −20.5, and **(F)** PK longitudinal base posterior: −22.2. A two-way repeated measures ANOVA followed by Fisher's LSD to determine differences among each experimental group and time point. *P*-values of <0.05 were considered statistically significant and indicated within each plot.

Conventional transthoracic echocardiography (TTE) was performed at 2 (TTE1), 5 (TTE2), and 8 (TTE3) weeks post-IR to longitudinally track the impact of PNA5 treatment on cardiac function and morphology post-IR. Two-way repeated-measures analysis implemented as a mixed-effects model revealed significant main effects of treatment (*p = 0.033) and time (*p = 0.035) on EF% in IR mice (Figure 2A). In sham mice, PNA5 treatment did not have any significant effect (*p* = 0.5181, two-way ANOVA; Supplementary Figure S1B). As expected, subjecting mice to the IR protocol significantly decreased EF% in both saline-treated and PNA5-treated groups when analyzed 2 weeks post-IR surgery (TTE1) compared with sham controls (Supplementary Figure S1B, Supplementary Table S1). Sham controls included saline-treated and PNA5-treated groups for up to 12 weeks and demonstrated no impact of long-term PNA5 administration as we have previously shown in Ang-(1-7)-treated mice (18, 19, 28).

By 5 weeks post-IR (TTE2), the EF% of PNA5-treated animals had significantly improved compared with that of saline-treated mice (TTE2) (*p* = 0.026, Fisher's LSD; Figure 2A) and approached the average EF% observed in sham animals. At 5 weeks post-IR, PNA5-treated mice had significantly improved values compared with PNA5-treated mice at 2 weeks post-IR (*p* = 0.024, Fisher's LSD) (Figure 2A). At 8 weeks post-IR, PNA5-treated mice maintained EF% levels that were not significantly different from 5-week EF% levels but also not different from 2-week levels or sham levels. Furthermore, EF% levels in PNA5-treated mice at 8 weeks were not significantly different from the EF% levels in saline-treated mice at 8 weeks. Saline-treated EF% remained significantly less post-IR compared with sham controls and unchanged over the 8-week treatment protocol.

EDV showed no significant change with time or treatment (Figure 2B). The ESV, however, significantly improved (**p = 0.0061, mixed-effects analysis) over the experimental protocol, which occurred more rapidly in the PNA5-treated group (**p = 0.0098at 2-weeks vs. 5-weeks, TTE1 vs. TTE2, Fisher's LSD) than in the saline-treated group (*p = 0.034at 2 weeks vs. 8 weeks, TTE1 vs. TTE3, Fisher's LSD) (Figure 2C). Together, these findings indicate that PNA5 preserves systolic function following IR injury and accelerates early functional recovery, while highlighting the need for complementary measures beyond conventional TTE to fully characterize therapeutic effects post-IR.

### PNA5 improves global longitudinal strain values

To further interrogate myocardial mechanics beyond conventional volumetric measures, STE-based strain imaging was used to assess the effects of PNA5 treatment following IR injury. Myocardial deformation in the longitudinal plane reflects a functional shortening of endocardial fibers. Global longitudinal strain (GLS) reflects myocardial deformation of endocardial fibers in the longitudinal plane and is an early indicator of cardiac dysfunction, especially following MI (13, 29). Similar to EF%, subjecting mice to the IR protocol significantly increased (worsened) GLS in both saline-treated and PNA5-treated groups when analyzed 2 weeks post-IR surgery (TTE1) compared with sham controls (Supplementary Figure S1C). There was a significant impact of PNA5 (**p = 0.0017, two-way ANOVA) on GLS that also improved over the experimental time course (***p = 0.0007, two-way ANOVA) (Figure 2D).

At 5 weeks post-IR (TTE2), GLS values in PNA5-treated mice significantly improved compared with the GLS values in these mice at 2-week post-IR (**p = 0.002, Fisher's LSD; Figure 2D). Furthermore, at 5 weeks post-IR, PNA5-treated mice had significantly improved GLS values compared with saline-treated mice (***p = 0.0009, Fisher's LSD). This improvement was maintained at 8 weeks post-IR (TTE3), with GLS values in PNA5-treated animals remaining significantly improved relative to TTE1 (*p = 0.024, Fisher's LSD) and significantly greater than saline-treated mice at TTE3 (**p = 0.006, Fisher's LSD). In contrast, saline-treated animals did not show the same improvements, also with no significant improvement over time. Together, these findings indicate that PNA5 accelerates and sustains improvement in GLS values following IR injury.

Left coronary ligation instigating the MI predominantly impacts perfusion to the anterior myocardium. Accordingly, we calculated the highest strain value or peak strain (PK%) at both the anterior and the posterior basal myocardium. PK% showed a similar pattern as GLS (Figure 2E) with important regional differences. There was a significant impact of PNA5 treatment on PK% at the base whether measured at the anterior (*p = 0.0174, two-way ANOVA) or posterior (**p = 0.0094, two-way ANOVA) region. Anterior PK% (Figure 2F) measured at the base showed a significant improvement (*p = 0.0497, Fisher's LSD) in the PNA5-treated group compared with the saline-treated group by 5 weeks post-IR (TTE2). The posterior myocardium also showed a significant benefit (**p = 0.0094, two-way ANOVA) with PNA5 treatment that showed significant differences over time (**p = 0.025, two-way ANOVA). This improvement in PK% with PNA5 was evident at 2 and 5 weeks post-IR (saline vs. PNA5 at TTE1: *p = 0.044 and saline vs. PNA5 at TTE2; *p = 0.025, Fisher's LSD). The posterior myocardium in saline-treated animals showed delayed recovery of PK%, which demonstrated significance at 8 weeks (TTE3) when compared with 2 weeks (TTE1; **p = 0.0101). Similar T2P trends were measured in the posterior midwall and posterior apex wall where T2P on the LV long axis was significantly higher in the PNA5-treated mice at 5 weeks post-IR compared with saline-treated mice (Supplementary Figures S2B,D). No significant changes were observed in the anterior walls (Supplementary Figures S2A,C). These data suggest that PNA5 not only prevents worsening strain but also promotes improved heart wall movement on the longitudinal axis.

### PNA5 reverses LV dyssynchrony

#### Longitudinal dyssynchrony

**Figure 3:**
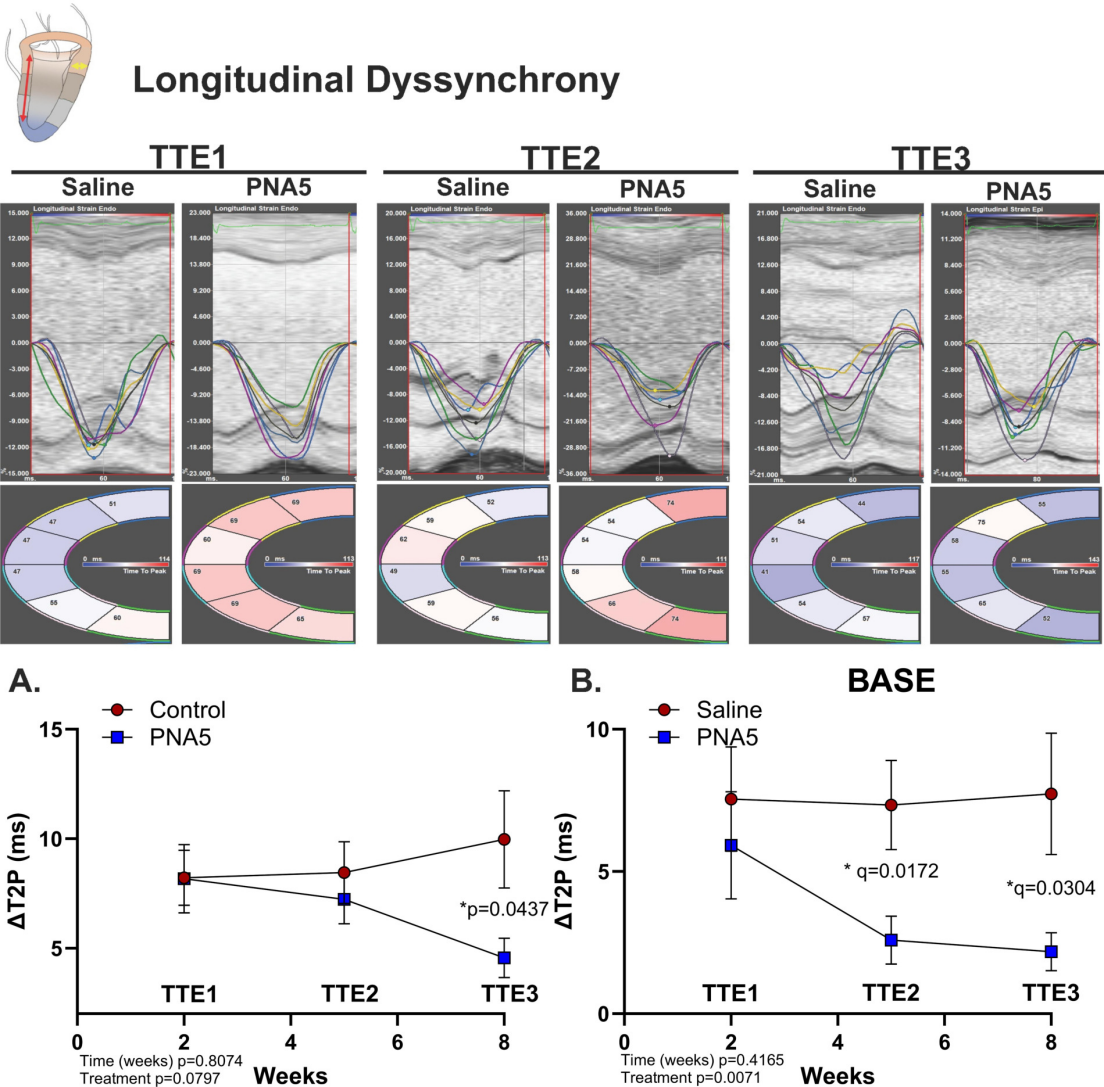
PNA5 protects against longitudinal dyssynchrony post-IR. Top panel: Segmental longitudinal synchronicity determined from longitudinal strain traces. Segments are color-coded with the corresponding strain (%) over time (ms) traces. Inset: A cartoon of cardiac PLAX view; the red arrow represents longitudinal strain during systole and the yellow arrow represents radial strain. Line plot representations of saline- (red circles) and PNA5 (blue squares)-treated mice at time points TTE1, TTE2, and TTE3 for **(A)** global longitudinal dyssynchrony and **(B)** basal longitudinal dyssynchrony determined by the time-delay-to-peak strain (ΔT2P; Saline, *n* = 12; PNA5, *n* = 13). A two-way repeated measures ANOVA followed by the two-stage Benjamini–Krieger–Yekutieli procedure (*Q* = 0.05) to determine differences among each experimental group and time point. *P*-values of <0.05 were considered statistically significant and indicated by * within each plot.

Global and regional differences in longitudinal dyssynchrony were measured to assess the heart's functional movement by investigating both the opposing-wall delay (ΔT2P) and regional heterogeneity (STD T2P). Regional reversal in longitudinal dyssynchrony following treatment was observed primarily at the base of the heart (**p = 0.0071, two-way ANOVA; Figure 3B). At the base of the heart PNA5-treated mice had a significant decrease in dyssynchrony by week 5 (TTE2; *q = 0.0172, two-stage Benjamini–Krieger–Yekutieli) compared with saline-treated mice that continued through week 8 (TTE3; *p = 0.0304, two-stage Benjamini–Krieger–Yekutieli). In contrast, no statistically significant global effect of treatment on longitudinal dyssynchrony was detected across the left ventricle (Figure 3A). These results indicate that PNA5 can improve longitudinal uniformity during systole following IR injury.

#### Radial dyssynchrony (long axis)

**Figure 4:**
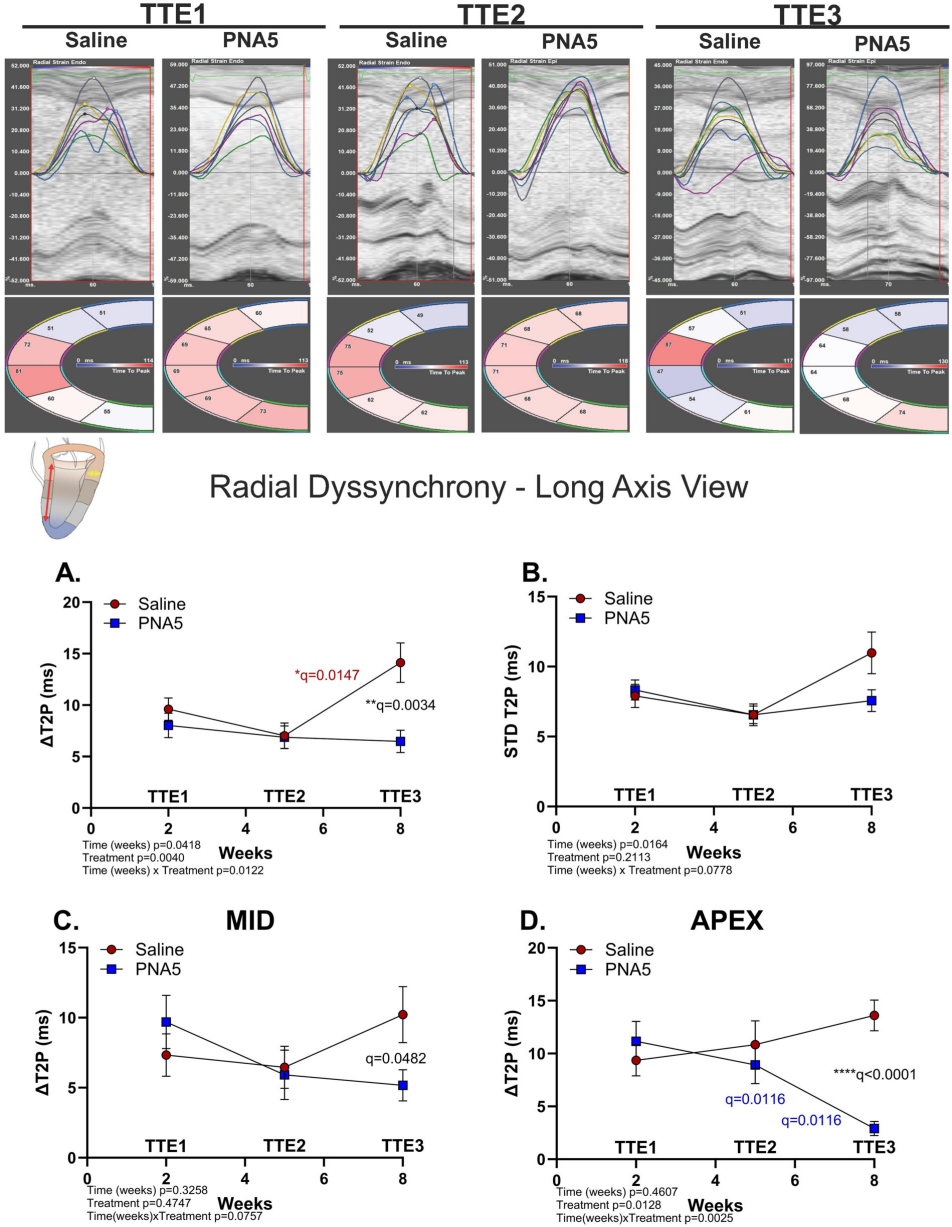
PNA5 protects against radial dyssynchrony post-IR (PLAX view). Top panel: Segmental radial synchronicity determined from the strain traces in the PLAX view. Segments are color-coded with the strain (%) over time (ms) traces. Inset: A cartoon of cardiac PLAX view; the red arrow represents longitudinal strain during systole, and the yellow arrow represents radial strain. Line plot representations of saline- (red circles) and PNA5 (blue squares)-treated mice at time points TTE1, TTE2, and TTE3 for **(A)** Radial dyssynchrony as ΔT2P (saline, *n* = 12; PNA5, *n* = 13) and **(B)** Radial dyssynchrony calculated as the standard deviation of T2P (STD T2P; saline, *n* = 12; PNA5, *n* = 14). Radial dyssynchrony as ΔT2P was measured at **(C)** the mid (saline, *n* = 12; PNA5, *n* = 13) and **(D)** apex(saline, *n* = 12; PNA5, *n* = 13). A two-way repeated measures ANOVA followed by a two-stage Benjamini–Krieger–Yekutieli procedure (*Q* = 0.05) was used to determine differences among each experimental group and time point. *P* values of <0.05 were considered statistically significant and indicated within each plot.

Because radial strain was measured from both the long axis and the short axis, we determined radial dyssynchrony from both parasternal long- and short-axes views. In the long-axis view, radial dyssynchrony (ΔT2P) revealed a significant treatment effect (**p = 0.0040, two-way ANOVA) that persisted over time (*p = 0.0418, two-way ANOVA), with a significant time × treatment interaction (*p = 0.0122, two-way ANOVA; Figure 4A). These effects were driven by a progressive worsening of radial dyssynchrony in saline-treated mice, which were significantly greater compared with the effects in PNA5-treated mice at 8 weeks (TTE3; **q = 0.0034, two-stage Benjamini–Krieger–Yekutieli) and in saline-treated mice at 5 weeks (TTE2; *q = 0.015, two-stage Benjamini–Krieger–Yekutieli).

Radial dyssynchrony calculated as the standard deviation of T2P (STD T2P) was significantly impacted over the time course of the protocol (*p = 0.0164, two-way ANOVA) but not by treatment independently (*p* = 0.2113, two-way ANOVA), with a trend toward a time × treatment interaction; (*p* = 0.0778; Figure 4B, two-way ANOVA).

In contrast, radial dyssynchrony measured at the midaxis level showed no significant difference over treatment or time (Figure 4C). However, radial dyssynchrony at the apex showed a more complex treatment responsive phenotype (Figure 4D). A mixed-effects analysis revealed that PNA5 had significant treatment effects (*p = 0.0128, two-way ANOVA) and a significant time × treatment interaction (**p = 0.0025, two-way ANOVA) for apical wall dyssynchrony, indicating that PNA5 significantly altered the temporal evolution of apical mechanical synchrony following IR injury. By 8 weeks post-IR, radial dyssynchrony at the apex had worsened in saline-treated mice but had attenuated in PNA5-treated mice. This was illustrated by the following significant differences: saline vs. PNA5 at TT3 (****q < 0.0001, two-stage Benjamini–Krieger–Yekutieli); PNA5 at TTE3 vs. TTE1 (**q = 0.0116, two-stage Benjamini–Krieger–Yekutieli); and TTE3 vs. TTE2 (*q = 0.0116, two-stage Benjamini–Krieger–Yekutieli).

#### Radial and circumferential dyssynchrony (short axis)

Radial and circumferential dyssynchrony assessed from the parasternal short-axis views quantify regional differences in time-to-peak strain, capturing temporal dispersion in myocardial thickening across the ventricular wall (radial strain) and circumferential shortening of the left ventricle (circumferential strain); these measures reflect the coordination of regional myocardial contraction following IR injury and are prognostic indicators of myocardial and LV recovery and remodeling post-IR (30). Consistent with long-axis measures of radial dyssynchrony, PNA5 treatment was associated with region-specific attenuation or improvement of systolic synchronization following IR.

**Figure 5:**
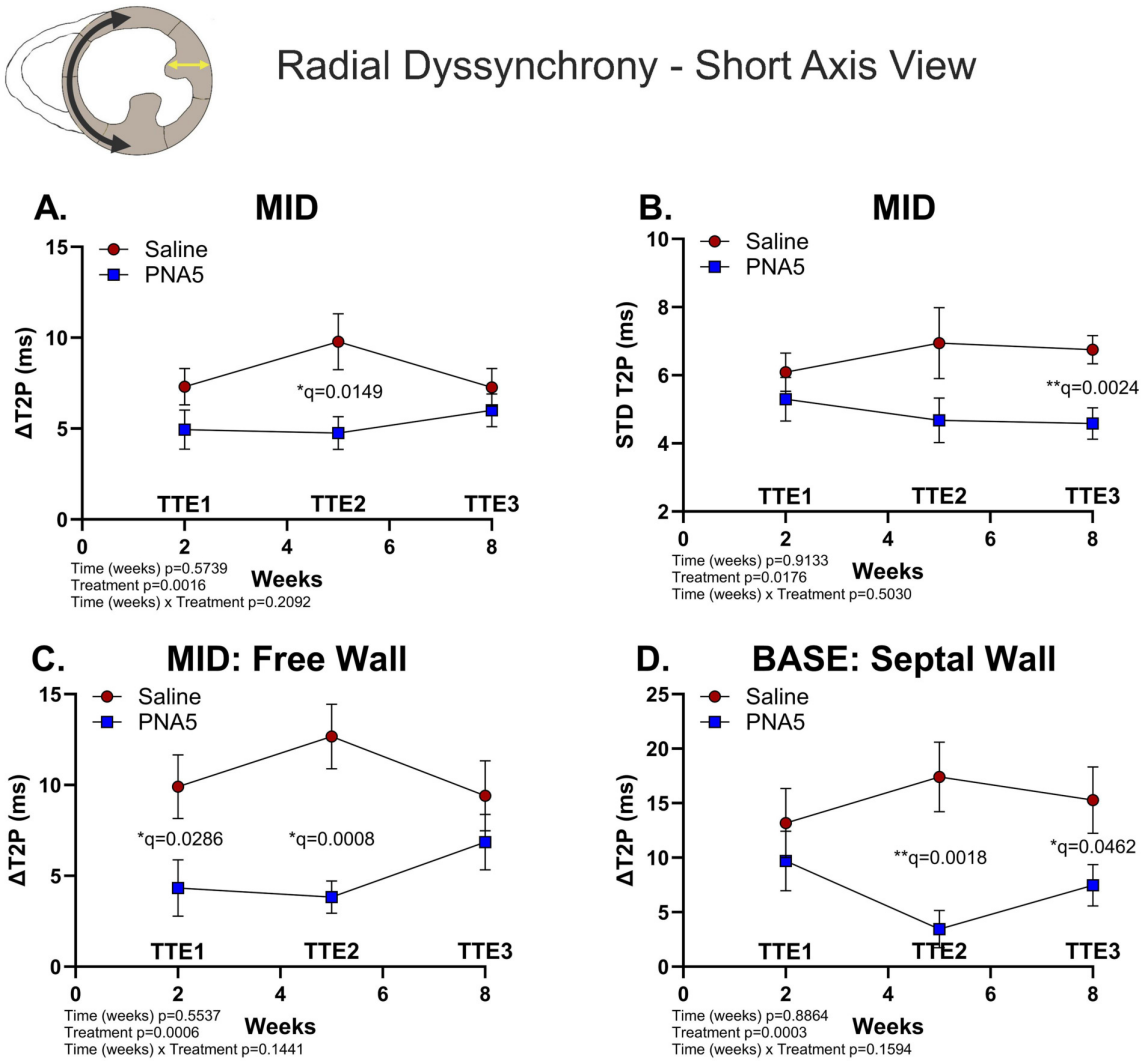
PNA5 protects against radial dyssynchrony post-IR (short-axis view). Segmental radial synchronicity determined from the short-axis view. Inset: A cartoon of cardiac short-axis view at the mid region; the black arrow represents circumferential strain during systole and the yellow arrow represents radial strain measured in the short-axis view. Line plot representations of saline- (red circles) and PNA5 (blue squares)-treated mice at time points TTE1, TTE2, and TTE3 for **(A)** radial dyssynchrony at midwall (MID) as ΔT2P and **(B)** radial dyssynchrony calculated as STD T2P; **(C)** radial dyssynchrony at the mid free wall (MID: Free Wall) as ΔT2P; **(D)** basal radial dyssynchrony at the septal wall (BASE: Septal) as ΔT2P (saline, *n* = 12; PNA5, *n* = 13). A two-way repeated measures ANOVA followed by the two-stage Benjamini–Krieger–Yekutieli procedure (*Q* = 0.05) was used to determine differences among each experimental group and time point. *P*-values of <0.05 were considered statistically significant and indicated within each plot.

Specifically, PNA5 treatment significantly impacted radial dyssynchrony measured in the short-axis at the midventricular region, as determined by the opposing-wall time-delay-to-peak strain (DT2P; **p = 0.0016, two-way ANOVA) marked by a significant difference between saline and PNA5 at 5 weeks post-IR (TTE2; *q = 0.0149, two-stage Benjamini–Krieger–Yekutieli; Figure 5A). PNA5 also significantly reduced regional heterogeneity of contraction timing, as assessed by the standard deviation of time-to-peak strain (STD T2P; *p = 0.0176, two-way ANOVA) marked by a significant difference between saline and PNA5 at 8 weeks post-IR (TTE3; **q = 0.0024, two-stage Benjamini–Krieger–Yekutieli; Figure 5B).

Specific regional differences in the mid and base short-axis planes showed beneficial treatment-dependent outcomes at both the mid free wall (ΔT2P, ***p = 0.0006, two-way ANOVA; Figure 5C) and the base septal wall (ΔT2P, ***p = 0.0003, two-way ANOVA; Figure 5D). At the mid free wall, PNA5-treated mice demonstrated early reductions in dyssynchrony relative to saline-treated mice at week 2 (TTE1; *q = 0.0286, two-stage Benjamini–Krieger–Yekutieli) and week 5 (TTE2; ***q = 0.0008, two-stage Benjamini–Krieger–Yekutieli), which were no longer evident by week 8 (TTE3). Septal dyssynchrony at the bae plane, in contrast, illustrated a benefit of PNA5 treatment over saline at week 5 (TTE2; **q = 0.0018, two-stage Benjamini–Krieger–Yekutieli) and week 8 (TTE3; *q = 0.0462, two-stage Benjamini–Krieger–Yekutieli) but not at week 2 (TTE1). Together, these data demonstrate that PNA5 treatments following IR attenuate radial dyssynchrony in the short axis at the mid and base sections of the heart.

**Figure 6:**
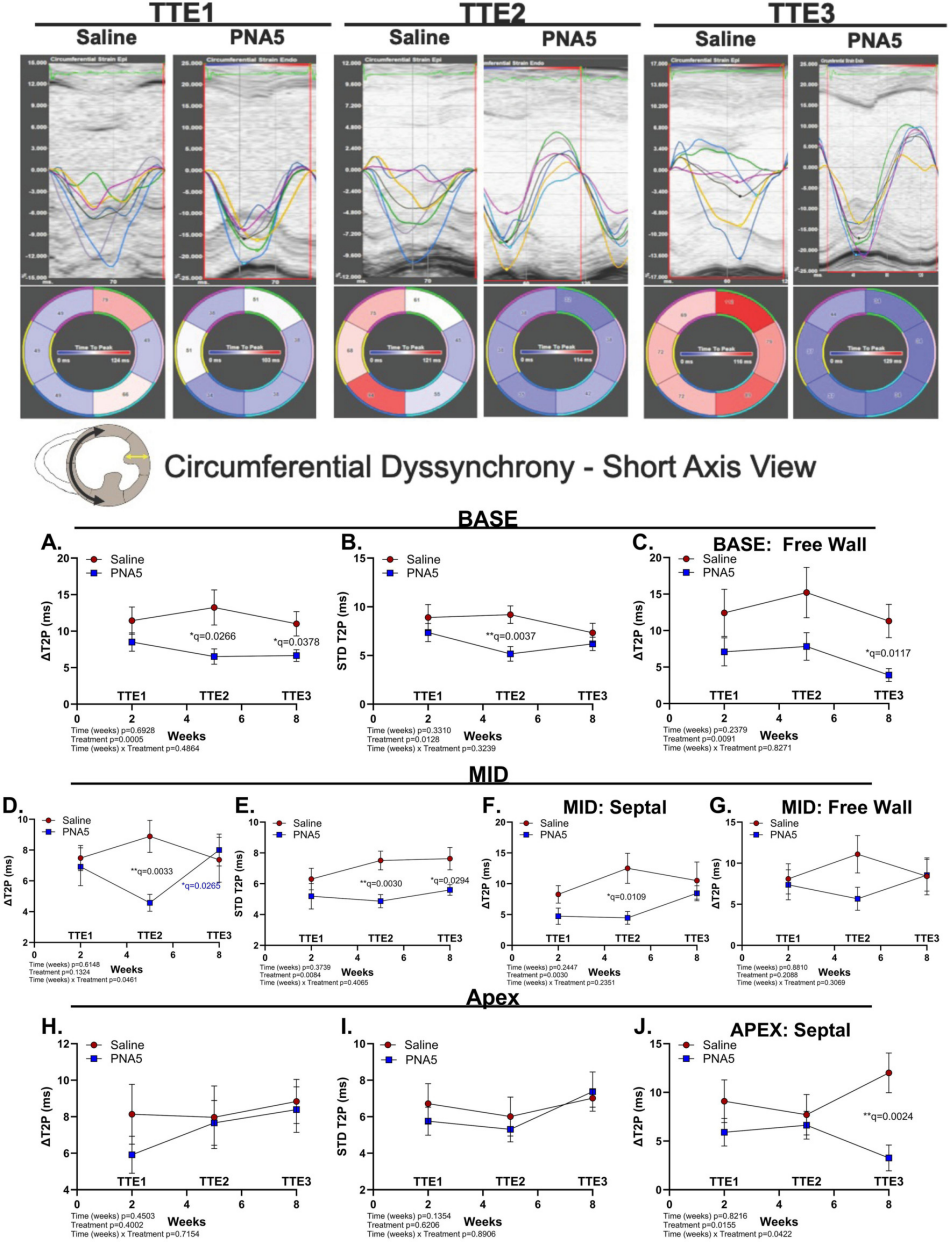
PNA5 protects against circumferential dyssynchrony post-IR. Top panel: Segmental circumferential synchronicity determined from train traces in the short-axis view. Segments are color-coded to correspond with the strain (%) over time (ms) traces. Inset: A cartoon of cardiac short-axis view at the mid region; the black arrow represents circumferential strain during systole and the yellow arrow represents radial strain measured in the short-axis view. Line plot representations of saline- (red circles) and PNA5 (blue squares)-treated mice at time points TTE1, TTE2, and TTE3 measured at the base (BASE), mid (MID), and apex (APEX) of the heart. Basal (BASE) circumferential dyssynchrony calculated as ΔT2P **(A)** and STD T2P **(B)** including basal circumferential dyssynchrony at the free wall region **(C)**. Mid (MID) circumferential dyssynchrony calculated as ΔT2P **(D)** and STD T2P **(E)** including mid circumferential dyssynchrony (ΔT2P) at the septal **(F)** and free wall regions **(G)** Apical (APEX) circumferential dyssynchrony calculated as ΔT2P **(H)** and STD T2P **(I)** including apical circumferential dyssynchrony (ΔT2P) at the septal **(J)** wall. (Saline, *n* = 12; PNA5, *n* = 13). A two-way repeated measures ANOVA followed by the two-stage Benjamini–Krieger–Yekutieli procedure (Q = 0.05) was used to determine differences among each experimental group and time point. *P*-values of <0.05 were considered statistically significant and indicated within each plot.

Although radial dyssynchrony is better correlated with LV remodeling post-IR, circumferential dyssynchrony may better predict regional wall shortening dyssynchrony as a reflection of circumferentially oriented fibers in the myocardium. Anatomically, treatment-related reductions in dyssynchrony appeared to follow a pattern from the free wall toward the septum spanning from the base toward the apex (Figure 6).

Circumferential opposing wall dyssynchrony at the BASE was significantly impacted by PNA5 treatment measured by time-delay-to-peak strain (DT2P; **p = 0.0005, two-way ANOVA; Figure 6A) driven by a significant difference between saline and PNA5 at TTE2 (*q = 0.0266, two-stage Benjamini–Krieger–Yekutieli) and TTE3 (*q = 0.0378, two-stage Benjamini–Krieger–Yekutieli). PNA5 also significantly reduced regional heterogeneity of contraction timing, as assessed by the standard deviation of time-to-peak strain (STP T2P; **p = 0.0128, two-way ANOVA) driven by a significant difference between saline and PNA5 at TTE2 (**q = 0.0037, two-stage Benjamini–Krieger–Yekutieli; Figure 6B). Specific regional opposing wall differences at the base were observed at the free wall. At the free wall (base), circumferential dyssynchrony was significantly impacted by PNA5 treatment (**p = 0.0091, two-way ANOVA; Figure 6C); PNA5-treated mice had significantly decreased dyssynchrony than saline-treated mice at 8 weeks (TTE3; *q = 0.0117, two-stage Benjamini–Krieger–Yekutieli).

PNA5 had a significant impact on circumferential opposing wall dyssynchrony at the MID plane over time (*p = 0.0461, two-way ANOVA; Figure 6D), indicated by an attenuation of dyssynchrony in PNA5-treated mice compared with their saline-treated counterparts at 5 weeks (TTE2; **q = 0.0033, two-stage Benjamini–Krieger–Yekutieli) that appeared to normalize by 8 weeks (TTE2 vs. TTE3; *q = 0.0265, two-stage Benjamini–Krieger–Yekutieli). Significantly reduced dyssynchrony measured by STD T2P was treatment-dependent (**p = 0.0084, two-way ANOVA; Figure 6E), with a significant difference between PNA5-treated mice and saline-treated mice by 5 weeks (TTE2; **q = 0.0030, two-stage Benjamini–Krieger–Yekutieli) that maintained through week 8 (TTE3; *q = 0.0294, two-stage Benjamini–Krieger–Yekutieli).

No significant treatment or time differences were observed when analyzing the total apex plan for either ΔT2P or STD T2P. Only regional treatment impact was observed in the apex. Differences in circumferential dyssynchrony at the apex were limited to the septal region (Figures 6H–J). PNA5 treatment resulted in significant differences in septal circumferential dyssynchrony in the mid (**p = 0.0030, two-way ANOVA; Figure 6F) and apex (*p = 0.0155, two-way ANOVA; Figure 6J) planes. In addition, on the apex plane, PNA5 treatment overtime had a significant impact (*p = 0.0422, two-way ANOVA; Figure 6J). Specifically, PNA5 attenuated midseptal circumferential dyssynchrony 5 weeks post-IR (TTE2; Saline vs. PNA5; *q = 0.0109, two-stage Benjamini–Krieger–Yekutieli; Figure 6F). Septal circumferential dyssynchrony at the apex showed that PNA5-treated mice demonstrated significant improvement by 8 weeks post-IR (TTE3; **q = 0.0024, two-stage Benjamini–Krieger–Yekutieli; Figure 6J).

### PNA5 improves cardiac outcomes post-IR

#### PNA5 attenuates infarct size

**Figure 7:**
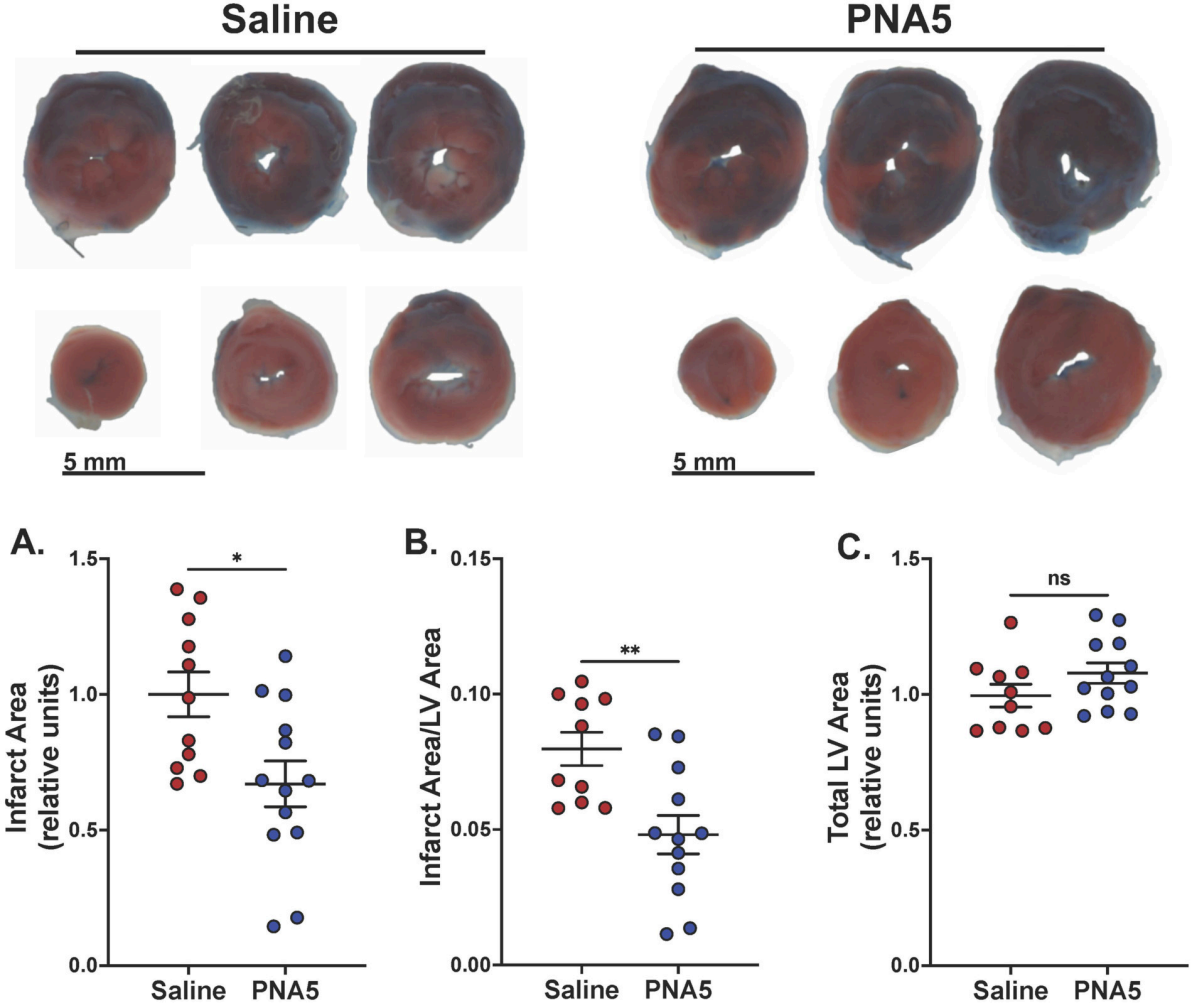
PNA5 attenuated infarct size post-IR. Top panel: Six serial sections of hearts from saline-treated and PNA5-treated mice 8 weeks post-IR identifying the area at risk (AAR) and infarct area in white as described in the Methods section (scale bar is 5 mm). **(A)** A scatter dot plot of the absolute infarct area in PNA5-treated hearts relative to saline-treated control hearts (*p = 0.0111). **(B)** A scatter dot plot of the absolute infarct area normalized to the total left ventricular area (infarct area/LV rea) in PNA5- and saline-treated hearts (**p = 0.0036). **(C)** The total LV Area determined from PNA5- and saline-treated hearts post-IR. (Saline, *n* = 12; PNA5, *n* = 13). An unpaired Student's *t*-test was used to determine differences among each experimental group and time point. *P*-values of <0.05 were considered statistically significant and indicated within each plot.

STE-based strain imaging measures myocardial deformation along multiple axes, permitting temporal and spatial evaluation of a myocardial infarction. In this study, strain analysis, coupled with calculations of dyssynchrony, indicated that PNA5 treatment impacted the size, function, and location of cardiac injury post-IR. Ligation of the left coronary artery was confirmed by visual blanching starting from the occlusion site to the apex of the anterior myocardium as described in the Methods sections. Following sacrifice, the hearts were excised, and the absolute infarct size was determined as a percentage of the entire LV indicated by the staining and histology (Figure 7; top panel). At 8 weeks post-IR, the PNA5 treatment significantly (*p = 0.0111) decreased the infarct area by 33.0% ± 8.4% compared with the saline treatment (Figure 7A). When the infarct size was normalized to the total LV area, the impact of the PNA5 treatment was amplified where PNA5-treated hearts showed a significant (**p = 0.0036) decrease in the infarct area normalized to the total LV area by 39.7% ± 8.9% compared with the saline treatment (Figure 7B). There was no impact of PNA5 on the total LV area (Figure 7C). These results indicated that PNA5 decreased damage to cardiac morphology following IR injury.

#### PNA5 decreases IR-related fibrosis

**Figure 8:**
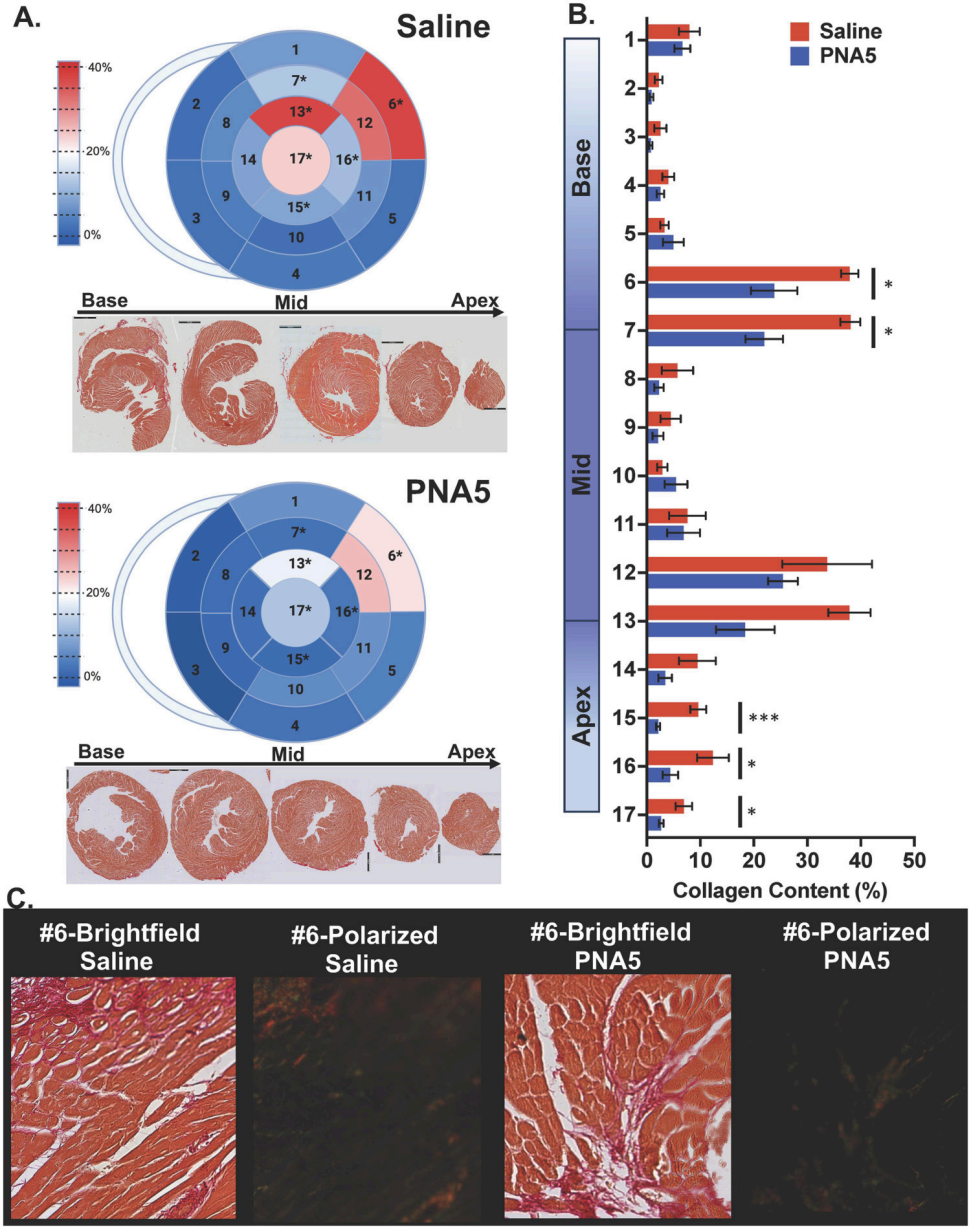
PNA5 attenuated fibrosis within specific LV segments post-IR. Left top panel: Bull’s eye representation in saline-treated and PNA5-treated hearts mapping 17 segments from the base to the apex of the heart image illustrating the regional distribution and quantity of fibrosis. A color gradient with associated key represents the percent fibrosis relative to the segment area. **(A)** Bright field image of 5–6 serial sections arranged from the base to mid to the apex of the heart. **(B)** A bar graph representation of segmental fibrosis content in saline-treated and PNA5-treated hearts. Significant differences are indicated as follows for segments 6 (*p = 0.042), 7 (*p = 0.013), 15 (***p = 0.0001), 16 (*p = 0.036), and 17 (* *p* = 0.018). A full list of segmental values and statistical differences is indicated in Table 2. Segmental comparisons between saline- and PNA5-treated hearts were performed using unpaired Student's *t*-tests for predefined LV regions. These analyses were hypothesis-driven; a value of *p* ≤ 0.05 was considered statistically significant; *n* = 4–6 for each group. **(C)** Representative zoomed-in brightfield images and underpolarized light of segment 6 from saline- (left panel) and PNA5-treated (right panel) hearts.

To quantify further morphological change, fibrosis of the left ventricle was measured using PSR staining. PNA5 decreased IR-related collagen inlay throughout the anterior and anterolateral areas of the heart (Figure 8, Supplementary Table S2). Bull’s eye plots of the mean percentage of collagen inlay reveal a decrease in fibrosis within the PNA5-treated heart compared with the IR-saline-treated heart primarily localized in segments that follow the infarct area of an occluded LAD (Figure 8; left panel). The bull’s eye represents the mean collagen% inlay, where red has the highest percentage of collagen with a maximum of 40%, white represents 20% collagen inlay, and blue represents 0% collagen inlay. Fibrosis appears to have increased toward the base of the heart with a greater amount around the infarct zones (the anterior and anterolateral walls). The percentage of collage inlay is represented as both a bar graph and a table (Figure 8; right panel). PNA5 decreased collagen inlay with a greater occurrence closer to the apex of the heart. However, collagen prevalence in the infarct area significantly decreased through the heart's base, mid, and apical segments. Specifically, there was a significant decrease in the percentage of collagen in the basal anterolateral, mid anterior, apical, anterior, apical inferior, apical lateral, and apex segments of the heart.

### PNA5 decreases IR-related inflammation in heart tissue

PNA5 significantly decreased TNFα (red) expression within the infarct areas of ischemic reperfusion injured hearts (*p = 0.0497, unpaired *t*-test; Supplementary Figures S3A,C). There were no changes in TNFα in the total LV area (Supplementary Figure S3B). These results suggested that PNA5 decreased inflammation in the infarct areas.

PNA5 also significantly decreased HIF1α at the infarct area hearts at 8 weeks post-IR (*p = 0.0398, unpaired *t*-test; Supplementary Figures S4A,C–F). There was no change in HIF1α in other regions of the heart including border distal areas from the infarct (Supplementary Figures S4B,C–F). These results suggested that PNA5 decreased HIF1α in the infarct areas.

### Global longitudinal strain correlates with infarct size and inflammation

In efforts to gain insight into how PNA5 improved GLS values post-IR, we correlated the values of infarct size to the GLS values. We found that global longitudinal strain significantly positively correlated with infarct size (Supplementary Figure S5), indicating that as the infarct size increases, GLS worsens.

### PNA5 protects memory impairment post-IR

When compared with mice that underwent sham surgeries, IR-saline-treated mice had a significantly decreased discrimination ratio, indicating cognitive impairment (*p = 0.0282, Tukey's multiple comparisons test; Supplementary Figure S6). PNA5 protects cognitive decline in IR mice as PNA5-treated mice have a significantly higher discrimination ratio than saline-treated mice (**p = 0.0028, Tukey's multiple comparisons test). These results suggest that IR injury can negatively impact memory. Furthermore, we observed that PNA5 protected against cognitive decline in IR mice as IR-PNA5-treated mice had a significantly higher discrimination ratio than IR-saline-treated mice.

## Discussion

Revascularization of ischemic tissue after MI is the most effective strategy to salvage the myocardium, reduce infarct size, and prevent heart failure (5–7), but paradoxically, it exacerbates injury through IR–associated oxidative stress, inflammation, and calcium dysregulation, ultimately promoting adverse remodeling and cardiac dysfunction. In the present study, we demonstrate that treatment with PNA5 attenuates IR injury by reducing infarct size, limiting inflammation and fibrosis, and preserving cardiac function. Within this study, these protective effects were detected using sensitive speckle tracking echocardiography (STE)–based measures of myocardial strain, highlighting regional and subclinical dysfunction not captured by conventional indices such as ejection fraction (1, 8, 31–33).

Previous work shows that the administration of Angiotensin-(1-7) [Ang-(1-7)] reduces infarct size following IR via Mas receptor (MasR) activation (34) and presumably through attenuation of ROS activity and restoration of intracellular Ca^2+^ homeostasis (35). Building upon this established cardioprotective pathway, our group developed PNA5, a novel synthetic glycopeptide MasR agonist derived from Ang-(1-) that exhibits a substantially extended half-life and improved pharmacokinetic stability. Similar to Ang-(1-7), PNA5 decreases ROS, inhibits proinflammatory cytokine production and fibrosis, and promotes vasodilation (17–19).

In addition to its cardioprotective properties, we have shown that PNA5 can preserve blood–brain barrier integrity and protect against cognitive impairment in models of vascular contributions to cognitive impairment and dementia (VCID) following myocardial injury (17–19). Together, these findings suggest that PNA5 may provide a dual therapeutic advantage over Ang-(1-7) alone by targeting both cardiac injury and downstream neurovascular consequences of MI. While cognitive outcomes were not a primary focus of this study, a limited assessment of recognition memory was included to contextualize the broader therapeutic potential of PNA5 in IR-related injury. In summary, our study showed that PNA5 reduced infarct size post-IR, inflammation, and fibrosis while minimizing cardiac dysfunction (17–19, 34, 35).

### PNA5 mitigated IR-associated progression of cardiac dysfunction

To model a potential therapeutic approach for PNA5, we initiated the delivery of PNA5 upon recovery from the IR procedure. Using echocardiography, we tracked cardiac function longitudinally until sacrifice at 8 weeks post-IR. PNA5 treatment in mice significantly improved cardiac function measured by EF at 5 weeks post-IR compared with IR saline treatment in mice. Previous studies showed that EF in mice following MI treated with Olmesartan, which increases Ang-(1-7) and ACEII activity, significantly increased compared with controls (20). However, cardiac repair and remodeling in untreated mice had normalized cardiac function measured by EF by 8 weeks post-IR compared with PNA5-treated mice.

Two-dimensional speckle tracking with strain analysis may represent a more sensitive non-invasive measure of myocardial dysfunction than ejection fraction (EF) (9, 10). Furthermore, global longitudinal strain (GLS) is validated as a sensitive prognostic indicator for predicting clinical outcomes and pathological remodeling in patients with acute MI (11). We demonstrated that GLS significantly improved with PNA5 treatment in mice at 5 weeks and persisted up to 8 weeks post-IR compared with untreated controls, which remained compromised throughout the 8-week time frame. In addition, compromised GLS for up to 42 days validates a successful IR protocol (1). GLS in untreated mice remained approximately compromised (∼15%) for greater than 42 days (up to 8 weeks), indicating a successful IR model. We further demonstrated that GLS significantly correlated with infarct size post-IR, further validating that GLS represents an important prognostic indicator.

The protective effect of PNA5 treatment was also evident by an attenuation of longitudinal dyssynchrony measured as the highest segmental strain value (PK%) and the maximum time-delay-to-peak systolic strain (Time to Peak; DT2P). Considering that ventricular dyssynchrony is an independent predictor of death or heart failure, the suggestion is that PNA5 may be efficacious at reducing these pathological outcomes (36). We also completed a more detailed interrogation of radial (long and short axis) and circumferential dyssynchrony. We further showed that PNA5 improved synchrony on all three axes (longitudinal, radial, and circumferential) with specific regional effects post-IR. In general, the most notable beneficial effects of PNA5 appear to occur primarily at the base in regions potentially bordering the infarct area. These results suggest that PNA5 addresses morphological changes in an IR heart in different areas as progression of damage and repair ensues following the initial ischemic insult.

### PNA5 decreases infarct size and infarct-associated fibrosis post-IR

We demonstrated for the first time that PNA5 attenuated infarct size post-IR presumably through MasR activation. MasR activation via Ang-(1-7) imparts cardioprotective effects by decreasing ROS, inhibiting proinflammatory cytokine production and fibrosis, and promoting vasodilation (17–19). Cardiac MI induces a proinflammatory response including ROS activation and releases proinflammatory cytokines such as IL-6, IL-1β, and TNFα, which themselves can activate apoptosis. While reperfusion of ischemic tissue is essential for improved clinical outcomes, upon reperfusion, an additional 25%–40% of cardiac cells die because of inflammation and ROS production and inflammatory responses (37). We have demonstrated previously that PNA5 via MasR activation inhibits ROS production and proinflammatory cytokines, suggesting a similar mechanism in this study (19). In support of this contention, we show that PNA5 decreases HIF-1α and TNFα by immunohistochemistry specifically in the infarct region but not in the border or remote zone. PNA5 may also improve vascular function in the ischemic heart, as shown in our recent paper, where PNA5 reverses deficits in brain neurovascular coupling and blood–brain barrier integrity while suppressing macrophage infiltration in our model of VCID (38).

These same proinflammatory pathways trigger macrophage and myofibroblast activation, leading to increased collagen deposition particularly within the infarct area and the tissue immediately surrounding the infarct (39). Although a necessary part of infarct healing and repair, inflammation and collagen deposition contribute to worsened cardiac function, as increases in collagen deposition, in turn, increases wall stiffness, impacting both relaxation and contractility (8, 40). In this study, we showed that PNA5 significantly reduced fibrosis within the left ventricle, measured by PSR. The main areas that had a significant decrease in fibrosis from PNA5 treatments were within the infarct area and immediately bordering the infarct. In the aforementioned study (38), PNA5 also suppressed microglia activation following VCID, which are the resident macrophages in the central nervous system, suggesting that PNA5 may contribute to fibrosis suppression, while accelerating infarct healing and repair.

### PNA5 protects memory impairment in mice with ischemic reperfusion injury

Lastly, that PNA5 rescues the cognitive impairment in our previously established model of HF-induced VCID (17–19, 41). In the current study, we provide further evidence that IR induces cognitive impairment, similar to findings from previous studies employing acute myocardial ischemia/reperfusion injury models (42). For the first time, we demonstrate the efficacy of PNA5 using an IR model where PNA5-treated mice have a significantly improved discrimination ratio compared with saline-treated mice. These results further highlight the beneficial application of PNA5 treatment immediately following an acute MI event.

## Limitations

The primary outcome from this study suggests that PNA5 and MasR activation represents an efficacious treatment strategy for cardiac MI and IR injury in addition to our well-established benefit for HF-induced cognitive impairment and VCID. While PNA5's cognitive protective effects have been validated through MasR activation via MasR antagonist A779, we have yet to validate that the beneficial outcomes of post-IR PNA5 treatments are specifically due to MasR activation. Future experiments incorporating MasR antagonism (e.g., A779) will be necessary to definitively establish receptor specificity underlying the observed cardiac benefits of PNA5.

The present study was designed to evaluate chronic post-IR remodeling and functional outcomes, using a similar 8-week timeline that aligns with our prior studies that investigated PNA5-protective effects in VCID HF-related cognitive impairment. However, future studies incorporating early post-IR morphological time points will be important to define acute injury responses, disease progression, and the timing of PNA5 therapeutic intervention. In addition, circulating biomarkers of cardiac injury and dysfunction, such as B-type natriuretic peptide and cardiac troponins, were not measured in this study. Inclusion of these biomarkers in future studies would further enhance the translational relevance of PNA5, allowing us to track cardiac status alongside imaging-based and histological outcomes throughout the study.

Furthermore, STE-based analysis should be used in a limited capacity. We provided a comprehensive picture of STE-based analysis, even though GLS remained the primary parameter that correlated with infarct size. STE, especially in mice, is subject to significant reproducibility and standardization issues because of the extremely high heart rates, motion artifacts, and interuser variability. Finally, cardiac disease and VCID are complex, integrative pathologies instigated by overlapping mechanisms. The use of a pluripotent, anti-inflammatory, anti-ROS agent such as PNA5 may provide mechanistic insight as well as a therapeutic path for the treatment of cardiac disease and HF-induced cognitive impairment.

## Data Availability

The original contributions presented in the study are included in the article/Supplementary Material, and further inquiries can be directed to the corresponding author.

## References

[1] Yang Y Schena GJ Wang T Houser SR. Postsurgery echocardiography can predict the amount of ischemia-reperfusion injury and the resultant scar size. Am J Physiol Heart Circ Physiol. (2021) 320:H690–8. 10.1152/ajpheart.00672.2020 33356964 PMC12151487

[2] Rich MW. Epidemiology, clinical features, and prognosis of acute myocardial infarction in the elderly. Am J Geriatr Cardiol. (2006) 15:7–11; quiz 12. 10.1111/j.1076-7460.2006.05273.x 16415640

[3] Tavazzi L. Clinical epidemiology of acute myocardial infarction. Am Heart J. (1999) 138:S48–54. 10.1016/S0002-8703(99)70320-0 10426859

[4] Lloyd-Jones D Adams R Carnethon M De Simone G Ferguson TB Flegal K Heart disease and stroke statistics—2009 update. Circulation. (2009) 119:e21–181. 10.1161/CIRCULATIONAHA.108.191261 19075105

[5] O'neill W Timmis GC Bourdillon PD Lai P Ganghadarhan V Walton J Jr. A prospective randomized clinical trial of intracoronary streptokinase versus coronary angioplasty for acute myocardial infarction. N Engl J Med. (1986) 314:812–8. 10.1056/NEJM198603273141303 2936956

[6] Hausenloy DJ Yellon DM. Myocardial ischemia-reperfusion injury: a neglected therapeutic target. J Clin Invest. (2013) 123:92–100. 10.1172/JCI62874 23281415 PMC3533275

[7] Neri M Riezzo I Pascale N Pomara C Turillazzi E. Ischemia/reperfusion injury following acute myocardial infarction: a critical issue for clinicians and forensic pathologists. Mediat Inflamm. (2017) 2017:1–14. 10.1155/2017/7018393 PMC5327760 28286377

[8] Schirone L Forte M D’ambrosio L Valenti V Vecchio D Schiavon S An overview of the molecular mechanisms associated with myocardial ischemic injury: state of the art and translational perspectives. Cells. (2022) 11:1165. 10.3390/cells11071165 35406729 PMC8998015

[9] Johnson C Kuyt K Oxborough D Stout M. Practical tips and tricks in measuring strain, strain rate and twist for the left and right ventricles. Echo Res Pract. (2019) 6:R87–98. 10.1530/ERP-19-0020 31289687 PMC6612062

[10] Duncan AE Alfirevic A Sessler DI Popovic ZB Thomas JD. Perioperative assessment of myocardial deformation. Anesth Analg. (2014) 118:525–44. 10.1213/ANE.0000000000000088 24557101 PMC4066464

[11] Otterstad JE Norum IB Ruddox V Le ACM Bendz B Munkhaugen J Prognostic impact of non-improvement of global longitudinal strain in patients with revascularized acute myocardial infarction. Int J Cardiovasc Imaging. (2021) 37:3477–87. 10.1007/s10554-021-02349-2 34327649 PMC8604850

[12] Lopez-Candales A Hernandez-Suarez DF. Strain imaging echocardiography: what imaging cardiologists should know. Curr Cardiol Rev. (2017) 13:118–29. 10.2174/1573403X12666161028122649 27799029 PMC5452148

[13] HOIT BD. Strain and strain rate echocardiography and coronary artery disease. Circ Cardiovasc Imaging. (2011) 4:179–90. 10.1161/CIRCIMAGING.110.959817 21406664

[14] Kostenis E Milligan G Christopoulos A Sanchez-FERRER CF Heringer-Walther S Sexton PM G-protein–coupled receptor Mas is a physiological antagonist of the angiotensin II type 1 receptor. Circulation. (2005) 111:1806–13. 10.1161/01.CIR.0000160867.23556.7D 15809376

[15] Santos RA Simoes e Silva AC Maric C Silva DM Machado RP de Buhr I Angiotensin-(1-7) is an endogenous ligand for the G protein-coupled receptor Mas—PubMed. Proc Natl Acad Sci U S A. (2003) 100:8258–63. 10.1073/pnas.1432869100 12829792 PMC166216

[16] Pinheiro SRVB Simões E Silva AC Sampaio WO De Paula RD Mendes EP Bontempo ED Nonpeptide AVE 0991 is an angiotensin-(1–7) receptor Mas agonist in the mouse kidney. Hypertension. (2004) 44:490–6. 10.1161/01.HYP.0000141438.64887.42 15326087

[17] Hay M Vanderah TW Samareh-Jahani F Constantopoulos E Uprety AR Barnes CA Cognitive impairment in heart failure: a protective role for angiotensin-(1-7). Behav Neurosci. (2017) 131:99–114. 10.1037/bne0000182 28054808 PMC6456812

[18] Hoyer-Kimura C Konhilas JP Mansour HM Polt R Doyle KP Billheimer D Neurofilament light: a possible prognostic biomarker for treatment of vascular contributions to cognitive impairment and dementia. J Neuroinflammation. (2021) 18:236. 10.1186/s12974-021-02281-1 34654436 PMC8520282

[19] Hay M Polt R Heien ML Vanderah TW Largent-Milnes TM Rodgers K A novel angiotensin-(1-7) glycosylated Mas receptor agonist for treating vascular cognitive impairment and inflammation-related memory dysfunction. J Pharmacol Exp Ther. (2019) 369:9–25. 10.1124/jpet.118.254854 30709867 PMC6413771

[20] Wang J He W Guo L Zhang Y Li H Han S The ACE2-ang (1–7)-Mas receptor axis attenuates cardiac remodeling and fibrosis in post-myocardial infarction. Mol Med Rep. (2017) 16:1973–81. 10.3892/mmr.2017.6848 28656296 PMC5561970

[21] Tyrankiewicz U Olkowicz M Skórka T Jablonska M Orzylowska A Bar A Activation pattern of ACE2/Ang-(1–7) and ACE/Ang II pathway in course of heart failure assessed by multiparametric MRI *in vivo* in Tgαq*44 mice. J Appl Physiol. (2018) 124:52–65. 10.1152/japplphysiol.00571.2017 28970203

[22] Mckinney AC Fattah C Loughrey MC Milligan G Nicklin AS. Angiotensin-(1–7) and angiotensin-(1–9): function in cardiac and vascular remodelling. Clin Sci. (2014) 126:815–27. 10.1042/CS20130436 24593683

[23] Danilo CA Constantopoulos E Mckee LA Chen H Regan JA Lipovka Y Bifidobacterium animalis subsp. lactis 420 mitigates the pathological impact of myocardial infarction in the mouse. Benefic Microbes. (2017) 8:257–69. 10.3920/BM2016.0119 PMC5815367 28409534

[24] Konhilas JP Sanchez JN Regan JA Constantopoulos E Lopez-Pier M Cannon DK Using 4-vinylcyclohexene diepoxide as a model of menopause for cardiovascular disease. Am J Physiol Heart Circ Physiol. (2020) 318:H1461–73. 10.1152/ajpheart.00555.2019 32383991 PMC7311698

[25] Helm RH Leclercq C Faris OP Ozturk C Mcveigh E Lardo AC Cardiac dyssynchrony analysis using circumferential versus longitudinal strain. Circulation. (2005) 111:2760–7. 10.1161/CIRCULATIONAHA.104.508457 15911694 PMC2396330

[26] Cheng A Helm RH Abraham TP. Pathophysiological mechanisms underlying ventricular dyssynchrony. EP Europace. (2009) 11:v10–4. 10.1093/europace/eup272 19861385

[27] Cerqueira MD Weissman NJ Dilsizian V Jacobs AK Kaul S Laskey WK Standardized myocardial segmentation and nomenclature for tomographic imaging of the heart. Circulation. (2002) 105:539–42. 10.1161/hc0402.102975 11815441

[28] Hoyer-Kimura C Konhilas J Sweitzer N Ryan L Hay M. Angiotensin-(1-7)/Mas receptor agonist: a disease modifying therapeutic for VCID protects cognitive function and inhibits serum neurofilament light protein. Alzheimers Dement. (2022) 18:e059909. 10.1002/alz.059909

[29] Lehner C Gehwolf R Tempfer H Krizbai I Hennig B Bauer H-C Oxidative stress and blood–brain barrier dysfunction under particular consideration of matrix metalloproteinases. Antioxid Redox Signaling. (2011) 15:1305–23. 10.1089/ars.2011.3923 PMC6464004 21294658

[30] Mollema SA Liem SS Suffoletto MS Bleeker GB Van Der Hoeven BL Van De Veire NR Left ventricular dyssynchrony acutely after myocardial infarction predicts left ventricular remodeling. J Am Coll Cardiol. (2007) 50:1532–40. 10.1016/j.jacc.2007.07.025 17936151

[31] Cardilo-Reis L Witztum JL Binder CJ. When monocytes come (too) close to our hearts. J Am Coll Cardiol. (2010) 55:1639–41. 10.1016/j.jacc.2009.11.068 20378084

[32] Panizzi P Swirski FK Figueiredo JL Waterman P Sosnovik DE Aikawa E Impaired infarct healing in atherosclerotic mice with Ly-6C(hi) monocytosis. J Am Coll Cardiol. (2010) 55:1629–38. 10.1016/j.jacc.2009.08.089 20378083 PMC2852892

[33] Nahrendorf M Swirski FK Aikawa E Stangenberg L Wurdinger T Figueiredo JL The healing myocardium sequentially mobilizes two monocyte subsets with divergent and complementary functions. J Exp Med. (2007) 204:3037–47. 10.1084/jem.20070885 18025128 PMC2118517

[34] Pachauri P Garabadu D Goyal A Upadhyay PK. Angiotensin (1–7) facilitates cardioprotection of ischemic preconditioning on ischemia–reperfusion-challenged rat heart. Mol Cell Biochem. (2017) 430:99–113. 10.1007/s11010-017-2958-4 28293875

[35] Xie JX Hu J Cheng J Liu C Wei X. The function of the ACE2/Ang(1-7)/Mas receptor axis of the renin-angiotensin system in myocardial ischemia reperfusion injury. Eur Rev Med Pharmacol Sci. (2022) 26:1852–9. 10.26355/eurrev_202203_28330 35363333

[36] Shin S-H Hung C-L Uno H Hassanein AH Verma A Bourgoun M Mechanical dyssynchrony after myocardial infarction in patients with left ventricular dysfunction, heart failure, or both. Circulation. (2010) 121:1096–103. 10.1161/CIRCULATIONAHA.109.863795 20176989

[37] Cadenas S. ROS and redox signaling in myocardial ischemia-reperfusion injury and cardioprotection. Free Radic Biol Med. (2018) 117:76–89. 10.1016/j.freeradbiomed.2018.01.024 29373843

[38] Hoyer-Kimura C Hay M Konhilas JP Morrison HW Methajit M Strom J PNA5, A novel Mas receptor agonist, improves neurovascular and blood-brain-barrier function in a mouse model of vascular cognitive impairment and dementia. Aging Dis. (2023) 29:1927–51. 10.14336/AD.2023.0928 PMC11272189 37815905

[39] Zhu L Wang Y Zhao S Lu M. Detection of myocardial fibrosis: where we stand. Front Cardiovasc Med. (2022) 9:926378. 10.3389/fcvm.2022.926378 36247487 PMC9557071

[40] Schirone L Forte M Palmerio S Yee D Nocella C Angelini F A review of the molecular mechanisms underlying the development and progression of cardiac remodeling. Oxid Med Cell Longevity. (2017) 2017:1–16. 10.1155/2017/3920195 PMC5511646 28751931

[41] Hay M Xue B Johnson RF Beltz TG Johnson AK. Angiotensin (1-7) and Mas receptor activation inhibits aldosterone induced ROS inflammatory responses in neurons of the paraventricular nucleus (PVN). FASEB J. (2013) 27:696.5. 10.1096/fasebj.27.1_supplement.696.5

[42] Evonuk KS Prabhu SD Young ME Desilva TM. Myocardial ischemia/reperfusion impairs neurogenesis and hippocampal-dependent learning and memory. Brain Behav Immun. (2017) 61:266–73. 10.1016/j.bbi.2016.09.001 27600185 PMC5511033

