# Supplementary material for: A novel angiotensin(1-7) agonist, PNA5, reduces ischemic reperfusion injury and cardiac dysfunction
Source: Front Cardiovasc Med. 2026 Mar 6;13:1769276. doi: 10.3389/fcvm.2026.1769276 (PMC13002834; doi:10.3389/fcvm.2026.1769276)
Supplement: Supplementary file 1 [file Datasheet1.docx]

**SUPPLEMENTAL METHODS AND RESULTS**

**A novel Angiotensin(1-7) agonist, PNA5, reduces ischemic reperfusion injury and cardiac dysfunction**

Christina Hoyer-Kimura Ph.D.^1^, Meredith Hay Ph.D.^1,2,6^, Methawasin Methajit Ph.D.^4^, Robin Polt Ph.D.^5^, Victoria Salcedo B.S.^1^, Joshua P. Fricks BSN^3^, Arian Piepho B.S.^1^, Maricela Pier B.S.^4^, Vito Marino B.S.^1^, and John P. Konhilas Ph.D.^1^

^1^Department of Physiology, The University of Arizona, Tucson, AZ 85724, USA

^2^Evelyn F. McKnight Brain Institute, The University of Arizona, Tucson, AZ 85724, USA

^3^College of Nursing, The University of Arizona, Tucson, AZ 85724, USA

^4^Department of Cellular and Molecular Medicine, The University of Arizona, Tucson, AZ 85724, USA

^5^Department of Chemistry and Biochemistry, The University of Arizona, Tucson, AZ 85724, USA

^6^ProNeurogen, Inc, Tucson, AZ, USA

Running Title: **PNA5A NOVEL THERAPEUTIC FOR CARDIAC ISCHEMIA-REPERFUSION**

**Corresponding Author:**

John P. Konhilas, Ph.D.

Department of Physiology

University of Arizona College of Medicine

1501 N Campbell Rd

Tucson, AZ 85724

Email: konhilas@arizona.edu

**Supplemental methods:**

**Sham experimental timelines**

Sham mice were treated with saline or PNA5 for EF% comparison: mice underwent sham surgery via left-sided thoracotomy at the fourth intercostal space. 8 weeks post-surgery, mice were treated with saline or PNA5 (1.0mg/kg dissolved in saline) daily subcutaneously for 21 days. Echocardiographs were measured at baseline prior to surgery and at 4-, 8-, and 12-weeks post-surgery.

Control mice used in NOR comparisons: Control mice underwent sham surgery that consisted of a left-sided thoracotomy at the fourth intercostal space. Sham mice were treated five weeks post-surgery with or DMSO (n=5) s.c. for 28 days.

**Immunofluorescent staining and Tyramide Signal Amplification (TSA)**

5μm thick heart sections were stained with TNF𝛂 (17590-1AP, Proteintech, Rosemont, IL) (Rabbit, IgG 1:100) overnight at 4°C on a shaker. The following day, sections were incubated in Anti-rabbit IgG, HRP-linked Antibody (1:200) (#7074, Cell Signaling, Danvers, MA) for 2 hours at room temperature. The staining was amplified via TSA amplification at room temperature for 10 minutes via CF®647 Tyramide (Ex/Em 650/665 nm, #96022, Biotium, Fremont, CA). Nuclei were stained for 1 minute at 1:300 with DAPI (ThermoFisher cat. 62248). Slides were imaged at 10x (TNFα, Cy5, 400ms exposure time; DAPI, 20ms exposure time) and stitched using the Leica DMI6000 scope and software. Mean fluorescent intensity was determined using ImageJ.

**Novel Object Recognition (NOR) test**

After 8 weeks of Saline or PNA5 treatments mice's cognition was measured using a novel object recognition test (NOR) test as described previously (Hay et al., 2017;Hay et al., 2019;Hoyer-Kimura et al., 2021;Hoyer-Kimura et al., 2023). We have previously demonstrated that at 8 weeks post-MI, exploration in cognitive tests was not impacted in MI mice receiving either saline or PNA5-treated animals.

A brief description: for two days mice were habituated to the testing arena, for 10 minutes per day. The learning phase follows habituation on the third day. During the learning phase, two identical objects are able to be explored for 6 minutes within the testing arena. Mice were returned to their home cages for two hours at the end of the learning phase. Following the two-hour period, mice were placed again into the testing arena for the memory testing phase. During the memory phase a novel object replaces one of the two identical objects. For two minutes mice were allowed to explore both the objects during the testing phase.

To minimize confounding factors, between testing phases and animals, the testing arena and objects were cleaned with 70% ethanol to prevent olfactory cues and testing was conducted under consistent lighting and environmental conditions. All behavioral analyses were performed by investigators blinded to treatment group.

*Analysis:* to score the mice recognition memory we calculate a discriminatory ratio (Discrimination ratio) by: Discrimination ratio= time spent exploring the novel object (t_novel_) minus time spent exploring the familiar object (t_familiar_) divided by the total exploration time (Hay et al., 2017;Hay et al., 2019):

**Equation 1**  Discrimination ratio= (t_novel_- t_familiar_)/ (total exploration time)

A positive Discrimination ratio is a result of a mouse interacting more with the novel object than the familiar object. Whie a negative Discrimination ratio is a result of mice spending more time with the familiar object than the novel object.

**Table S1: Summary of conventional transthoracic echocardiography parameters.** Values are represented as mean ± SEM (n=12-13 for each group). Baseline sham values are presented for visual comparison. A 2-way repeated measures ANOVA followed by multiple comparisons analysis via Fisher's LSD to determine differences among each experimental group (saline, and PNA5) at each timepoint (TTE1, TTE2, TTE3). P values of <0.05 were considered statistically significant. Ejection fraction- EF, fractional shortening- FS, end-diastolic volume- EDV, end-systolic volume- ESV.

**Table S1**

|  | ***Baseline***  ***Sham*** |  | **Saline**  **TTE1** | **PNA5**  **TTE1** | ***P value***  ***TTE1*** |  | **Saline**  **TTE2** | **PNA5**  **TTE2** | ***P value***  ***TTE2*** |  | **Saline**  **TTE3** | **PNA5**  **TTE3** | ***P value***  ***TTE3*** |
| --- | --- | --- | --- | --- | --- | --- | --- | --- | --- | --- | --- | --- | --- |
| **EF**  **(%)** | *57.2±1.3* |  | 45.9±2.0 | 47.4±1.4 | *0.5709* |  | 47.4±2.5 | 56.2±2.6 | *0.0263** |  | 48.1±1.4 | 51.7±1.9 | *0.2857* |
| **EDV**  **(μL)** | *65.7±5.5* |  | 58.9±1.5 | 63.6±2.4 | *0.1146* |  | 54.1±1.7 | 58.6±3.0 | *0.2139* |  | 60.7±3.8 | 59.9±3.3 | *0.8693* |
| **ESV**  **(μL)** | *28.4±3.1* |  | 31.1±1.0 | 33.7±1.9 | *0.9681* |  | 28.5±1.5 | 25.0±2.7 | *0.0879* |  | 25.1±1.9 | 29.3±2.6 | *0.9915* |

**Table S2: Values for left ventricular segment collagen content.**Values for Saline- or PNA5-treated IR mice as representative of the ratio of pixels measuring area collagen content to area tissue for corresponding segments ranging from the Base to the apex.. Rows in in grey indicate measurements of collagen in an identifiable infarct within each segment. Values are represented as mean ± SEM (n=12-13 for each group). T-tests were used to compare PNA5 and Saline groups; P values of <0.05 were considered statistically significant.

**Table S2**

|  | **Segment** |  | **Saline** |  | **PNA5** |  | **P Value** |
| --- | --- | --- | --- | --- | --- | --- | --- |
| **1.** | **Basal Anterior** |  | 8.0±1.9 |  | 6.6±1.5 |  | 0.599 |
|  | *Basal Anterior (Infarct)* |  | *69.5±11.9* |  | *89.8±3.2* |  | *0.165* |
| **2.** | **Basal Anteroseptal** |  | 2.2±0.7 |  | 0.8±0.3 |  | 0.106 |
| **3.** | **Basal Inferoseptal** |  | 2.5± 1.1 |  | 0.7±0.3 |  | 0.125 |
| **4.** | **Basal Inferior** |  | 4.0±1.1 |  | 2.5±0.8 |  | 0.256 |
| **5.** | **Basal Inferolateral** |  | 3.3± 0.8 |  | 5.0±2.0 |  | 0.479 |
| **6.** | **Basal Anterolateral** |  | 37.9±1.6 |  | 23.8±4.3 |  | 0.042* |
|  | *Basal Anterolateral (Infarct)* |  | *98.6± 2.0* |  | *59.7±12.8* |  | *0.043** |
| **7.** | **Mid Anterior** |  | 14.2±3.5 |  | 3.7±1.1 |  | 0.013* |
|  | *Mid Anterior (Infarct)* |  | *91.4±9.5* |  | *55.2±10.2* |  | *0.027** |
| **8.** | **Mid Anteroseptal** |  | 5.7±3.0 |  | 2.3±0.9 |  | 0.287 |
| **9.** | **Mid Inferoseptal** |  | 4.5±1.9 |  | 2.1±1.0 |  | 0.429 |
| **10.** | **Mid Inferior** |  | 2.9±1.0 |  | 5.5±2.1 |  | 0.362 |
| **11.** | **Mid Inferolateral** |  | 7.6±3.4 |  | 6.9± 3.1 |  | 0.876 |
| **12.** | **Mid Anterolateral** |  | 33.7±8.4 |  | 25.4±2.8 |  | 0.34 |
|  | *Mid Anterolateral (Infarct)* |  | *74.3±18.5* |  | *55.7±4.5* |  | *0.352* |
| **13.** | **Apical Anterior** |  | 37.9±3.9 |  | 18.4±5.5 |  | 0.018* |
|  | *Apical Anterior (Infarct)* |  | *86.4±7.9* |  | *45.3±14.3* |  | *0.034** |
| **14.** | **Apical Septal** |  | 9.4±3.4 |  | 3.4±1.3 |  | 0.108 |
| **15.** | **Apical Inferior** |  | 9.6±1.5 |  | 2.1±0.4 |  | 0.001* |
| **16.** | **Apical Lateral** |  | 12.4±3.0 |  | 4.4±1.4 |  | 0.036* |
| **17.** | **Apex** |  | 24.2±1.9 |  | 13.7±2.9 |  | 0.018* |
|  | *Apex (Infarct)* |  | *96.6±6.9* |  | *57.6±15.1* |  | *0.056* |

**Supplemental Figure Legends:**

**Figure S1: PNA5 does not impact cardiac function measured by conventional and speckle tracking-based strain echocardiography.** (**S1A**) Ejection Fraction (EF%) over the course of 12 weeks in **Sham+Saline** and **Sham+PNA5**. Significance was tested by 2-way ANOVA followed by an uncorrected Fisher's LSD. (**S1B**) Scatter dot plot of measured EF% comparing baseline Sham controls (white circles) to both Saline- (red circles) and PNA5- (blue circles) treated mice post-IR at TTE1. (**S1C**) Scatter dot plot comparing Global longitudinal strain (**GLS**) in baseline Sham controls (white circles) to both Saline- (red circles) and PNA5- (blue circles) treated mice post-IR at TTE1. Significance for **S1B** and **S1C** were determined using one-way ANOVA with Tukey's multiple comparisons test; p-values ≤ 0.05 were considered significant.

**Figure S2: PNA5 protects against longitudinal dyssynchrony post-IR measured by time to peak strain.** Line plot representation showing time to peak strain (T2P, ms) of Saline- (red circles) and PNA5- (blue squares) treated mice at timepoints **TTE1**, **TTE2**, and **TTE3** in the following segments, MID: Anterior (**S2A**), MID: Posterior (**S2B**), APEX: Anterior (**S2C**), APEX: Posterior (**S2D**). A 2-way repeated measures ANOVA followed by Fisher's LSD to determine differences among each experimental group and timepoint. P values of <0.05 were considered statistically significant indicated within each plot.

**Figure S3: PNA5 decreased inflammatory marker, TNFɑ, post-MI.** (**S3A**): Scatter dot plot of mean fluorescence intensity of Tumor Necrosis Factor alpha (TNFɑ) in the infarct area. (**S3B**): Scatter dot plot of mean fluorescence intensity of TNFɑ in the total LV area. (**S3C**) Representative images show Saline (**left panel**) and PNA5 (**right panel**). DAPI-stained nuclei appear blue (20ms exposure). TNFɑ appears red (Cy5; 400ms exposure time). Images were taken at 10X. The scale bar is 2000µm. Significance was determined with students t-test, p-values ≤ 0.05 were considered significant.

**Figure S4: PNA5 decreased inflammatory marker, Hif1ɑ, post-MI.** (**S4A**): Scatter dot plot of mean fluorescence intensity of Hypoxia Inducible Factor 1 alpha (Hif1ɑ) in the infarct area. (**S4B**): Scatter dot plot of mean fluorescence intensity of Hif1ɑ in the total LV area. (**Bottom panel**) Representative images show Saline (**left panel; S4C,E**) and PNA5 (**right panel; S4D, F**). DAPI-stained nuclei appear blue (20ms exposure). Hif1ɑ appears red (Cy5; 400ms exposure time). Images were taken at 10X. The scale bar is 2000µm. Significance was determined with students t-test, p-values ≤ 0.05 were considered significant.

**Figure S5: Global longitudinal strain correlated with total infarct area.** (**S5A**)**:**correlation between infarct size and global longitudinal strain (GLS) are shown over the course of TTE1 (2-weeks post-IR), TTE 2 (5-weeks post-IR), and TTE3 (8-weeks post-IR). Simple linear regression was used to determine correlation and significance. P-values ≤ 0.05 were considered significant.

**Figure S6: PNA5 reverses cognitive impairment, post-MI.** Cognitive function was tested by a novel object recognition test (NOR) as a discrimination ratio for control (0.178±0.091, n=5) and mice with ischemic reperfusion (IR) mice. IR mice were treated with either saline (-0.410±0.123, n=11) or PNA5 (0.236±0.139, n=10). The discrimination ratio is the measurement of cognitive abilities to recognize new objects from previously learned objects. A positive discrimination ratio is indicative of intact cognitive abilities. A negative discrimination ratio or a decreased discrimination ratio is indicative of cognitive impairment. IR mice treated with saline are significantly decreased compared to both control mice (p=0.0282) and IR-PNA5 treated mice (p=0.0028). Significance was tested via one-way ANOVA, followed by Tukey's multiple comparisons tests. P values <0.05 are considered significant.

**References**

Hay, M., Polt, R., Heien, M.L., Vanderah, T.W., Largent-Milnes, T.M., Rodgers, K., Falk, T., Bartlett, M.J., Doyle, K.P., and Konhilas, J.P. (2019). A Novel Angiotensin-(1-7) Glycosylated Mas Receptor Agonist for Treating Vascular Cognitive Impairment and Inflammation-Related Memory Dysfunction. *J Pharmacol Exp Ther* 369**,** 9-25.

Hay, M., Vanderah, T.W., Samareh-Jahani, F., Constantopoulos, E., Uprety, A.R., Barnes, C.A., and Konhilas, J. (2017). Cognitive impairment in heart failure: A protective role for angiotensin-(1-7). *Behav Neurosci* 131**,** 99-114.

Hoyer-Kimura, C., Hay, M., Konhilas, J.P., Morrison, H.W., Methajit, M., Strom, J., Polt, R., Salcedo, V., Fricks, J.P., Kalya, A., and Pires, P.W. (2023). PNA5, A Novel Mas Receptor Agonist, Improves Neurovascular and Blood-Brain-Barrier Function in a Mouse Model of Vascular Cognitive Impairment and Dementia. *Aging and disease* Volume. 15**,** 25.

Hoyer-Kimura, C., Konhilas, J.P., Mansour, H.M., Polt, R., Doyle, K.P., Billheimer, D., and Hay, M. (2021). Neurofilament light: a possible prognostic biomarker for treatment of vascular contributions to cognitive impairment and dementia. *J Neuroinflammation* 18**,** 236.
