# Supplementary figures and images for: A novel angiotensin(1-7) agonist, PNA5, reduces ischemic reperfusion injury and cardiac dysfunction
Source: Front Cardiovasc Med. 2026 Mar 6;13:1769276. doi: 10.3389/fcvm.2026.1769276 (PMC13002834; doi:10.3389/fcvm.2026.1769276)

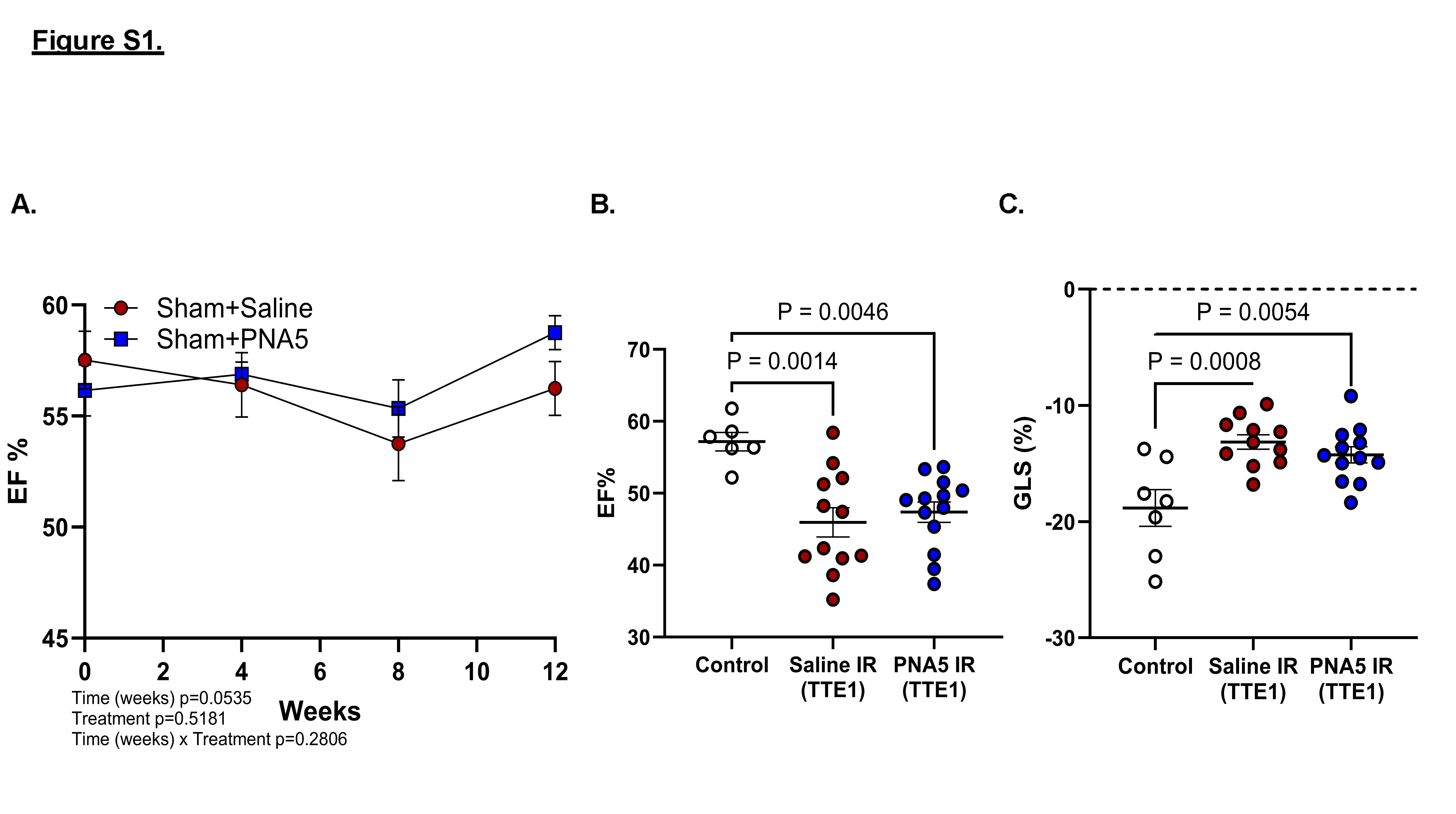

Supplement: Supplementary file 2 [file Image1.jpeg]

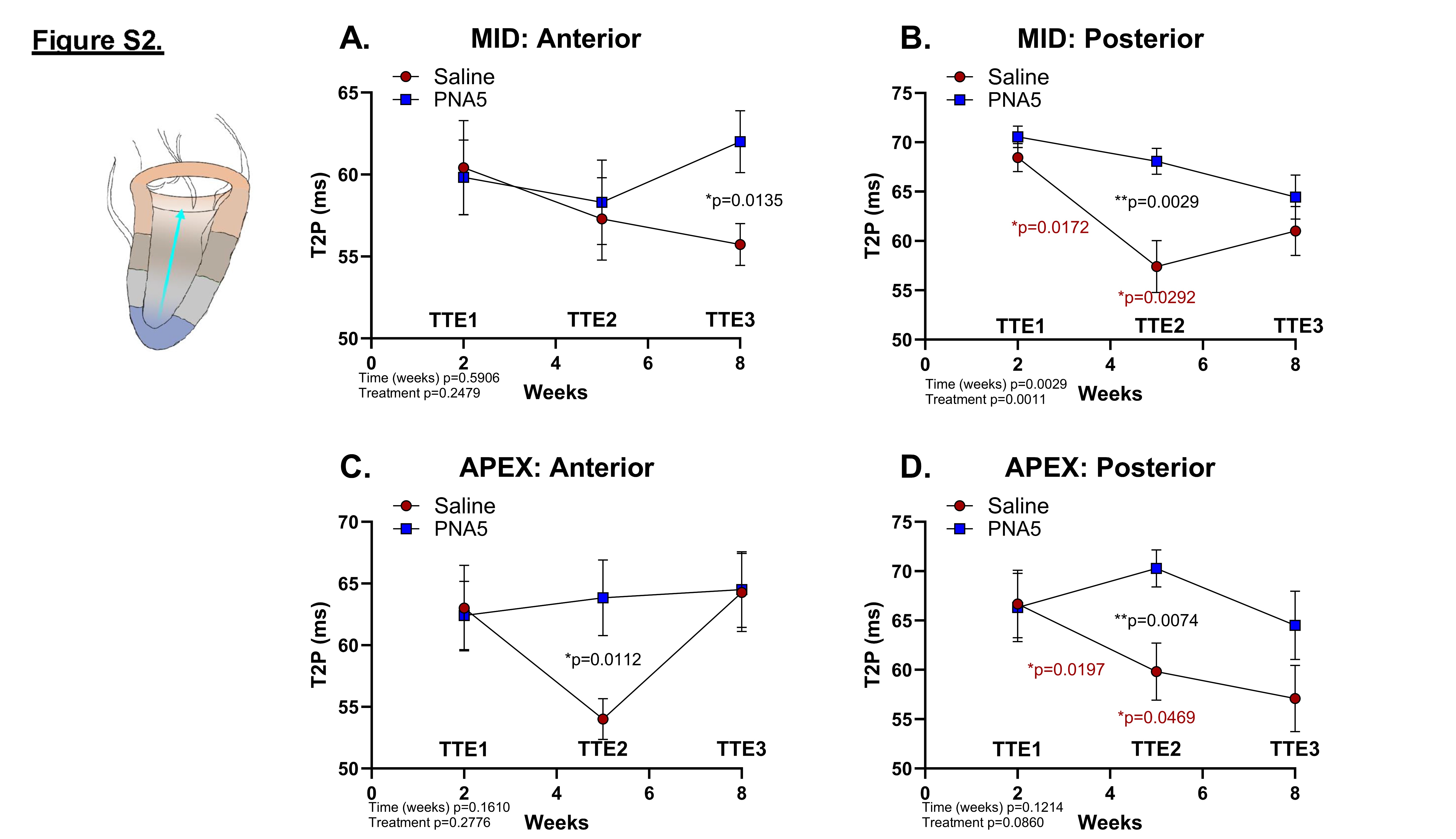

Supplement: Supplementary file 3 [file Image2.jpeg]

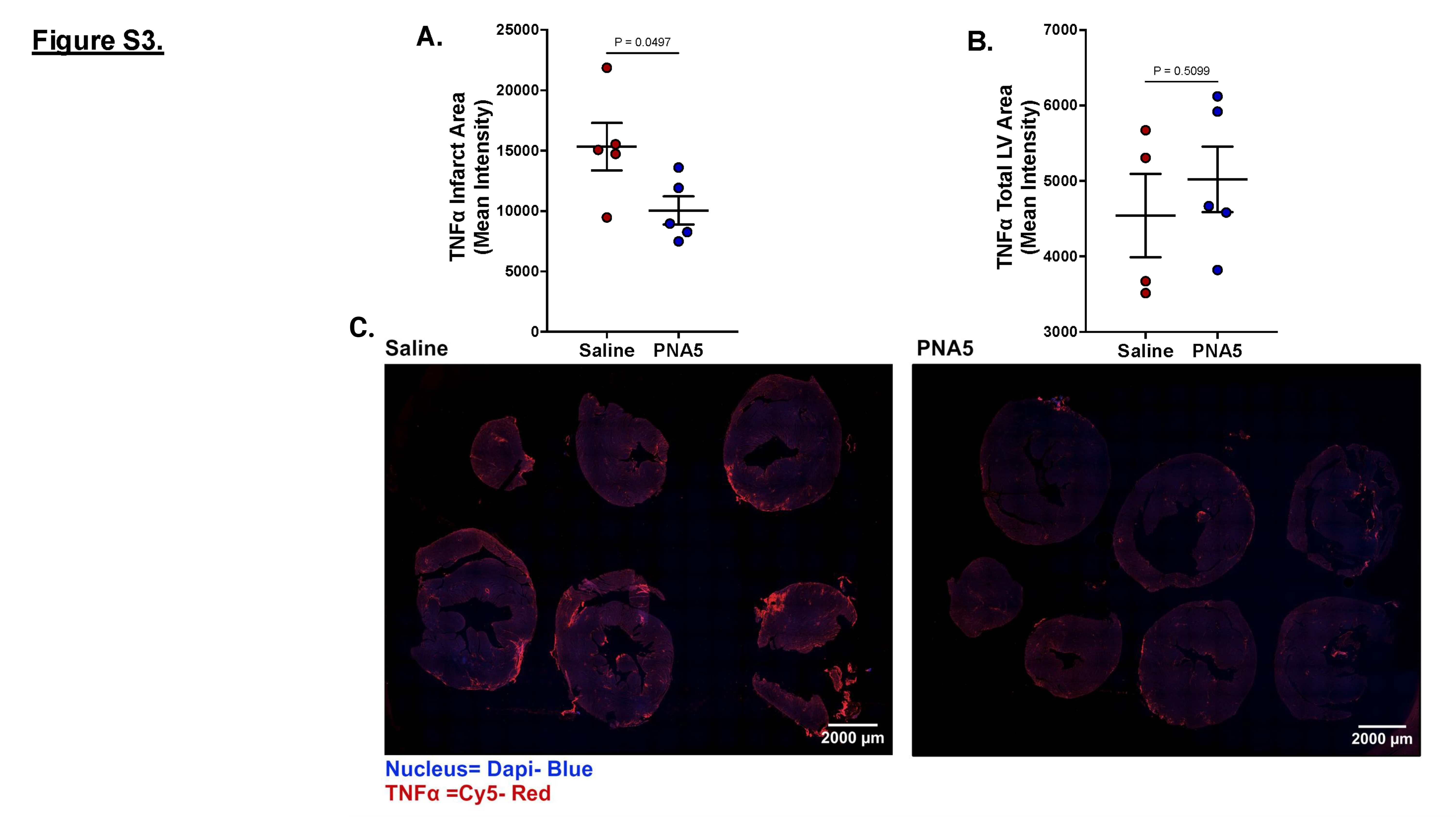

Supplement: Supplementary file 4 [file Image3.png]

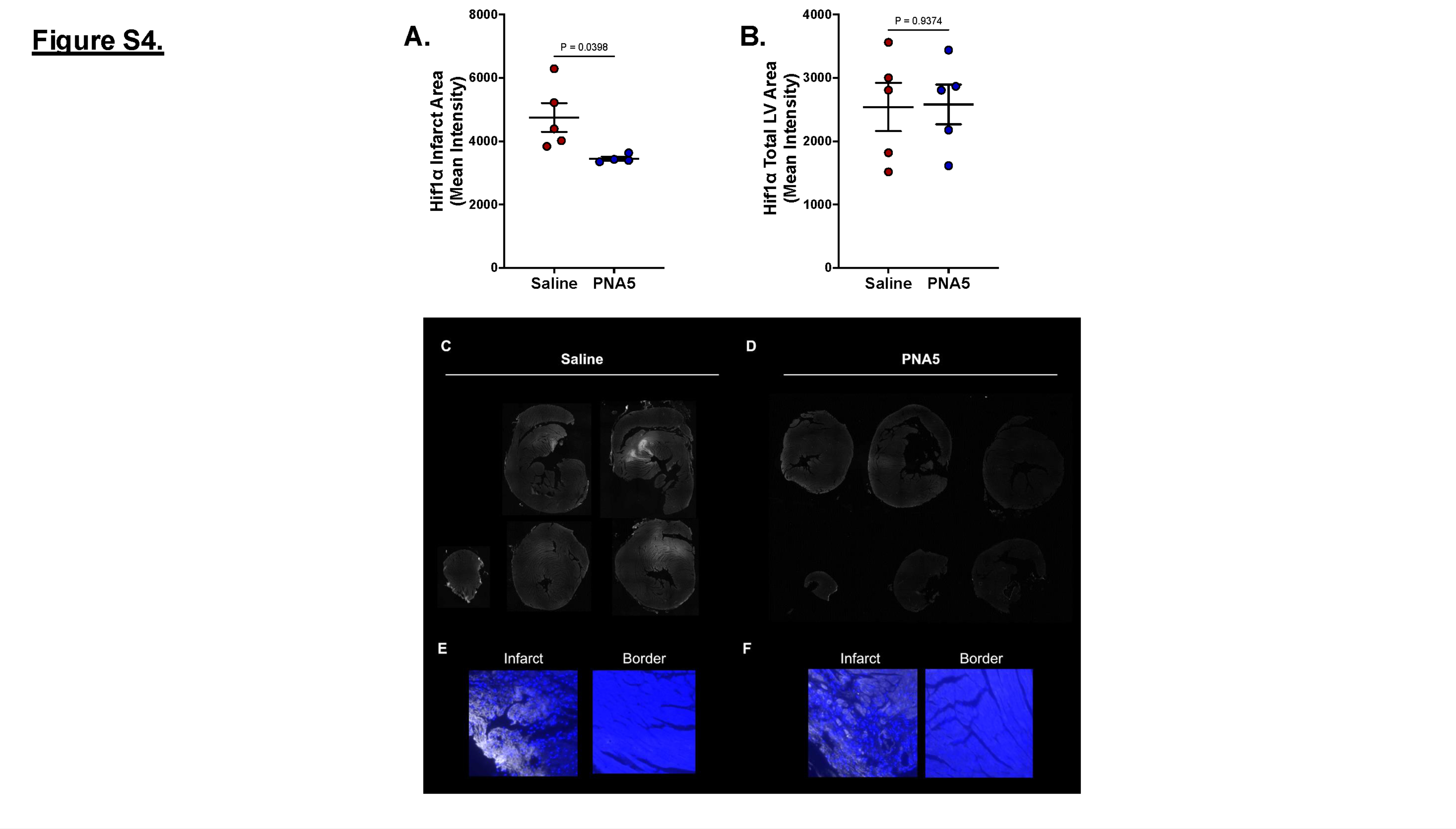

Supplement: Supplementary file 5 [file Image4.jpeg]

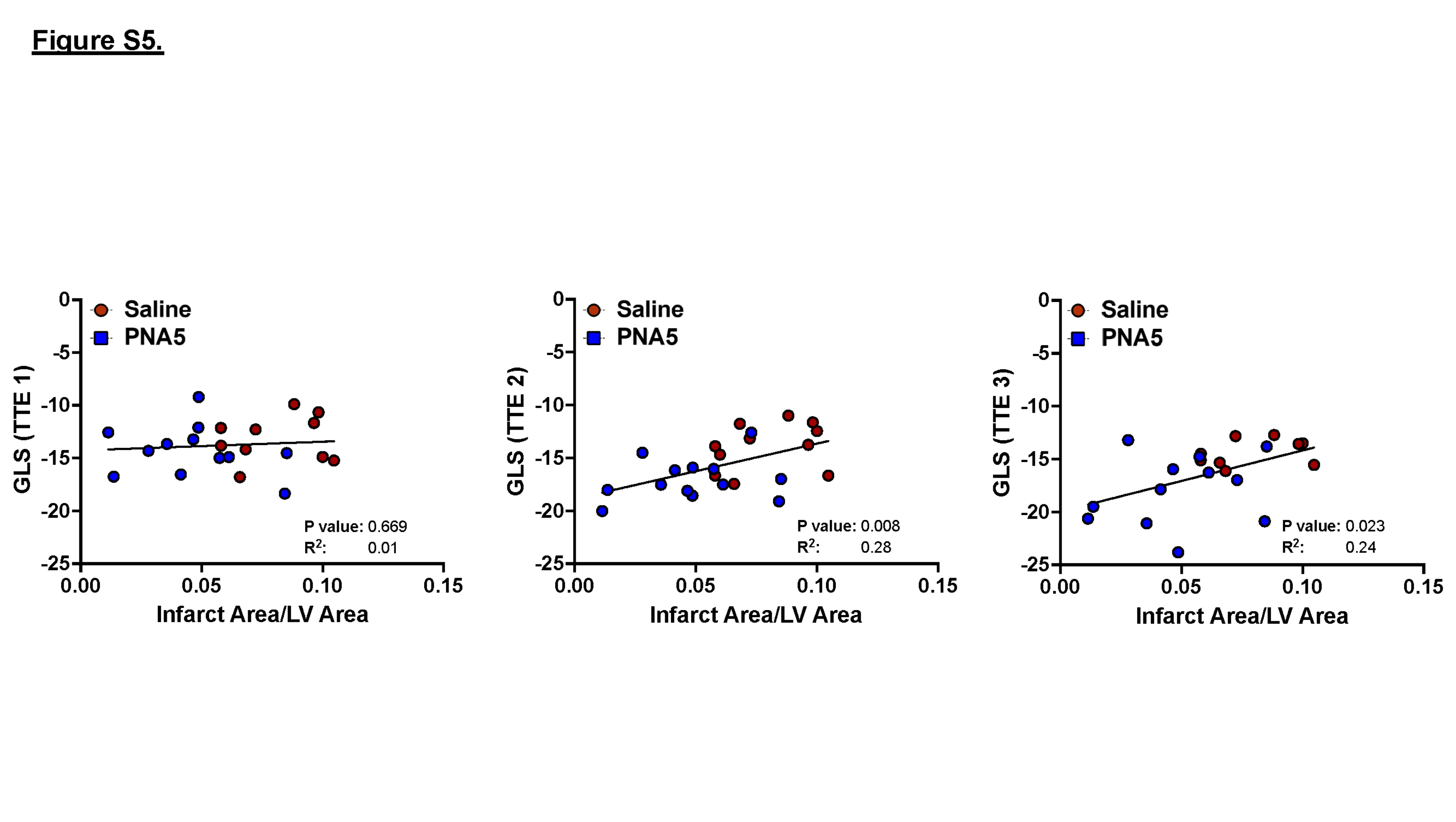

Supplement: Supplementary file 6 [file Image5.jpeg]

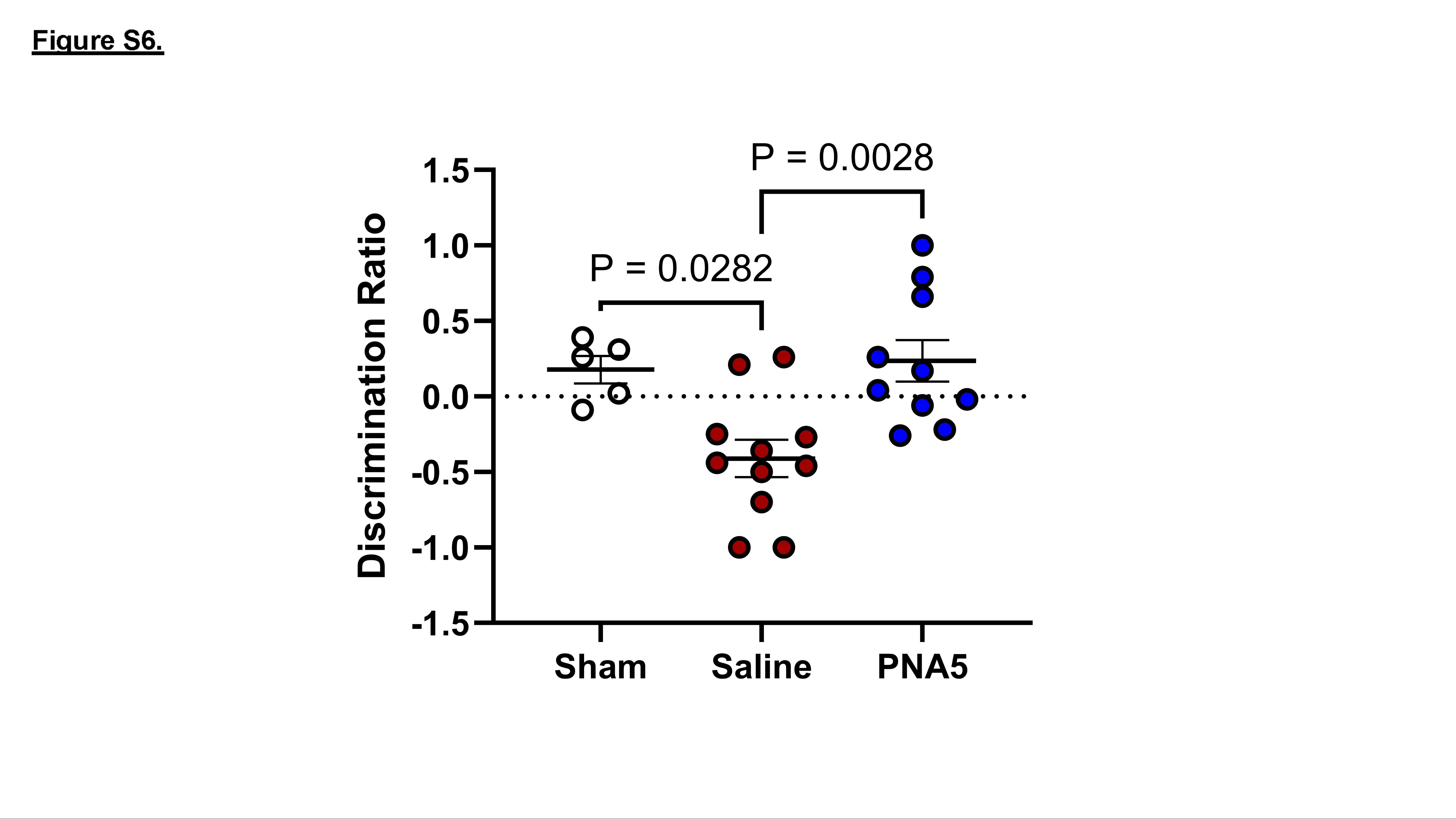

Supplement: Supplementary file 7 [file Image6.jpeg]
